# A novel histochemistry assay to assess and quantify focal cytochrome c oxidase deficiency

**DOI:** 10.1002/path.5084

**Published:** 2018-05-14

**Authors:** Marie‐Lune Simard, Arnaud Mourier, Laura C Greaves, Robert W Taylor, James B Stewart

**Affiliations:** ^1^ Max Planck Institute for Biology of Ageing Cologne Germany; ^2^ CNRS, Université de Bordeaux Institut de Biochimie et Génétique Cellulaires UMR5095, Bordeaux France; ^3^ Newcastle University LLHW Centre for Ageing and Vitality Newcastle University Newcastle upon Tyne UK; ^4^ Wellcome Centre for Mitochondrial Research, Institute of Neuroscience Newcastle University Newcastle upon Tyne UK; ^5^ NHS Highly Specialised Mitochondrial Diagnostic Laboratory Newcastle upon Tyne Hospitals NHS Foundation Trust Newcastle upon Tyne UK

**Keywords:** mitochondrial diseases, COX deficiency, cytochrome c oxidase, enzyme histochemistry, nitrotetrazolium blue chloride

## Abstract

Defects in the respiratory chain, interfering with energy production in the cell, are major underlying causes of mitochondrial diseases. In spite of this, the surprising variety of clinical symptoms, disparity between ages of onset, as well as the involvement of mitochondrial impairment in ageing and age‐related diseases continue to challenge our understanding of the pathogenic processes. This complexity can be in part attributed to the unique metabolic needs of organs or of various cell types. In this view, it remains essential to investigate mitochondrial dysfunction at the cellular level. For this purpose, we developed a novel enzyme histochemical method that enables precise quantification in fresh‐frozen tissues using competing redox reactions which ultimately lead to the reduction of tetrazolium salts and formazan deposition in cytochrome c oxidase‐deficient mitochondria. We demonstrate that the loss of oxidative activity is detected at very low levels – this achievement is unequalled by previous techniques and opens up new opportunities for the study of early disease processes or comparative investigations. Moreover, human biopsy samples of mitochondrial disease patients of diverse genotypic origins were used and the successful detection of COX‐deficient cells suggests a broad application for this new method. Lastly, the assay can be adapted to a wide range of tissues in the mouse and extends to other animal models, which we show here with the fruit fly, Drosophila melanogaster. Overall, the new assay provides the means to quantify and map, on a cell‐by‐cell basis, the full extent of COX deficiency in tissues, thereby expending new possibilities for future investigation. © 2018 The Authors. *The Journal of Pathology* published by John Wiley & Sons Ltd on behalf of Pathological Society of Great Britain and Ireland.

## Introduction

Defects in mitochondrial oxidative metabolism lead to human diseases with a broad clinical spectrum. Symptoms can manifest early or late in life and while neuromuscular problems are most common, any organ or tissue can be affected 1, 2, 3. This variability in the clinical manifestations remains largely unexplained and great efforts have been put into deciphering what causes this broad phenotypic expression and tissue specificity 2.

A powerful approach to investigate the state of the respiratory chain activity is the use of mitochondrial enzyme histochemistry. This allows the visualization of mitochondrial activity without disturbing the structural integrity of the tissue. By the 1980s, histochemical techniques had already identified focal mitochondrial Complex IV (cytochrome *c* oxidase or COX) deficiency in diseases due to pathogenic, nuclear‐encoded genes affecting mitochondrial function 4 or in normal ageing of human hearts 5. Later, it was found that clonal expansion of mitochondrial (mt) DNA mutations with age leads to COX deficiency in many human tissues, and in vertebrate animal models of ageing 6. Enzyme histochemical techniques have an advantage in visualizing single cell dysfunction, compared with other biochemical measurements of tissue homogenates where the averaging of biochemical competence across individual cells can mask cell‐specific mitochondrial defects. Quantitative analysis of COX activity has been described by several groups, where the oxidation of 3,3'‐diaminobenzidine (DAB) catalysed by COX is monitored using microphotometric assays 7, 8, 9. However, one drawback with this approach is the difficulty in distinguishing between pathogenic loss of COX activity versus cells with lower levels of COX activity in tissues where cells display heterogeneous mitochondrial activity 10. Alternatively, deficient COX activity can be revealed using a double‐labelling method known as COX/SDH (cytochrome *c* oxidase/succinate dehydrogenase) histochemistry 11. The COX/SDH method cleverly reveals cells with normal mitochondrial function in brown, through the oxidation of DAB, while cells with deficiencies in COX activity are revealed in blue. However, this dual colour hinders the detection of low or intermediate deficiency levels, whose light blue colour is obstructed by the predominant brown pigments; therefore, detection and quantification of COX deficiency remain incomplete 12.

Immunofluorescence techniques have also been developed to quantify the presence of several subunits of the respiratory chain complexes in tissues 13, 14, 15. These analyses enable simultaneous evaluation of multiple respiratory complexes in a single cell and are semi‐quantitative, providing sensitive detection of protein levels. The downsides of this approach are the costs and effort of developing species‐specific antibody sets, and the detection of protein rather than a measurement of enzyme function.

Here, we present a new enzyme histochemical assay that exploits the competing redox reactions between phenazine methosulphate (PMS), cytochrome *c* oxidase, and nitrotetrazolium blue chloride (nitro blue tetrazolium; NBT). In 1989, Old and Johnson described for a microphotometric assay, optimal protocols for the histochemical assay of both SDH and COX activity 7. Their work was subsequently confirmed in mouse tissues 16. In addition, literature dating back to the 1950s describes the use of PMS for the reduction of NBT 17, 18, as well as the interaction of reduced PMS with Complex IV 19, 20, 21, 22. Building upon these previous studies, we uncovered that the dual affinity of PMS for either NBT or Complex IV can be exploited to reveal COX‐deficient cells without the use of DAB and sequential reactions. Indeed, PMS is a strong electron carrier that transfers electrons from Complex II to NBT; but in a complete system where mitochondrial COX activity is not inhibited, PMS preferentially passes on electrons to molecular oxygen via electron acceptors located in the COX subunits 21, 22, 23. We show here that the alternative COX/SDH histochemical method – hereafter named nitrotetrazolium blue exclusion assay (NBTx) – can efficiently reveal low levels of COX deficiency in single cells. The strength of this new assay is through the catalysis of formazan only where COX activity is dysfunctional, leading to the direct visualization of respiratory‐deficient cells rather than a reduction in the predicted COX activity. The NBTx assay offers the unique opportunity to measure and quantify COX deficiency directly and unambiguously.

## Materials and methods

### Ethics statement

All animal work was approved by the Landesamt für Natur, Umwelt und Verbraucherschutz Nordrhein‐Westfalen in accordance with German and European Union regulations (Permits 84‐02.042015.A103; 84‐02.05.50.15.004). The use of human tissues in this study followed the ethical guidelines issued by the Newcastle and North Tyneside Local Research Ethics Committees (reference 09/H0906/75 and REC ref No 2001/188) and complied with the Declaration of Helsinki.

### Preparation of incubation media

Phosphate‐buffered saline (PBS; 0.1 m, pH 7.0) was prepared by mixing sodium dihydrogen orthophosphate monohydrate (0.04 m) with disodium hydrogen phosphate anhydrous (0.06 m).

NBTx solution contained 5 mg of NBT (Cat. No N6876; Merck, Darmstadt, Hesse, Germany) dissolved in 3.2 ml of PBS, to which thawed stock solutions of PMS (Cat. No P9625; Merck; 100 μl/ml, 2.0 mm in purified water) and sodium succinate (Cat. No S2378; Merck; 100 ul/ml, 1.3 M in 0.1 M PBS) were added. Final concentrations were 130 mM sodium succinate, 2 mM NBT, and 0.2 mM PMS, pH 7.0.

### NBTx assay

#### Tissue preparation

Small pieces of heart tissue were quickly frozen in 2‐methylbutane in a glass beaker cooled by immersion in liquid nitrogen. Frozen tissues were stored at −80 °C until ready to use. Testes and colons were frozen in Tissue‐Tek® O.C.T. compound (supplied by A Hartenstein GmbH, Wuerzburg, Germany) immersed in liquid nitrogen. Brain tissues were frozen slowly on dry ice. Heart tissues were cut at 7 μm, whereas brains, skeletal muscles, testes, and colons were cut at 10 μm using a cryostat at −20 °C (OFT 5000; Bright Instruments, Luton, UK), mounted on Superfrost Plus microscope slides (Menzel, Thermo Scientific, Waltham, MA, USA), and air‐dried for 5–10 min. Slides were kept at −80 °C for at most a few months to avoid loss of enzyme activity.

#### Staining protocol

Slides were taken out of the −80 °C freezer and thawed briefly at room temperature on a slide holder without a lid. Sections (three sections per slide) were then covered with 1 ml of PBS for 10 min in an incubator set at the desired temperature (1 ml per slide). PBS was discarded and replaced with 1 ml of NBTx solution. Incubation time and temperature varied according to tissue type. Generally, the best results were obtained with heart sections at 18 °C for 30 min. Brain, skeletal muscles, and testes required a higher incubation temperature (22–25 °C). Sections were then washed briefly in purified water and dehydrated in an ethanol series (2 min in 50%, 75%, 96%, and 100%, followed by an extra 5 min in 100% ethanol). Finally, slides were immersed for 5 min in two changes of xylene before being mounted on coverslips with Cytoseal™ (Thermo Scientific, Darmstadt, Germany).

### Inhibiting the respiratory chain

Sodium azide and potassium cyanide were dissolved in water, malonate in 1 m sodium hydroxide and all the other inhibitors in absolute ethanol at the following concentrations: TTFA (1 mm), atpenin A5 (0.1 mm), sodium azide (100 mm), potassium cyanide (100 mm), rotenone (1 mm), antimycin A (1 mm), myxothiazol (0.2 mm), oligomycin (3 mm), and malonate (6 m). Stock solutions were stored at −20 °C and thawed shortly before use. Inhibitors were added to PBS for the preincubation time as well as to the NBTx solution (rotenone, oligomycin, antimycin A, 1:500; myxothiazol, atpenin A5, TTFA, 1:200; sodium azide, potassium cyanide, 1:100; malonate, 1:1000). Control conditions contained 5 μl/ml of 100% ethanol or 1 μl/ml of 1 m NaOH accordingly. All conditions retained a neutral pH value between 6.9 and 7.2.

## Results

### NBTx assay

The NBTx assay involves only one colour (blue formazan crystals from NBT) and is based on strong biochemical competition between COX and NBT for the acquisition of electrons from PMS (see reaction scheme in Figure 2D). In COX‐competent mitochondria, minimal or no catalysis of formazan takes place and cells remain colourless.

**Figure 1:**
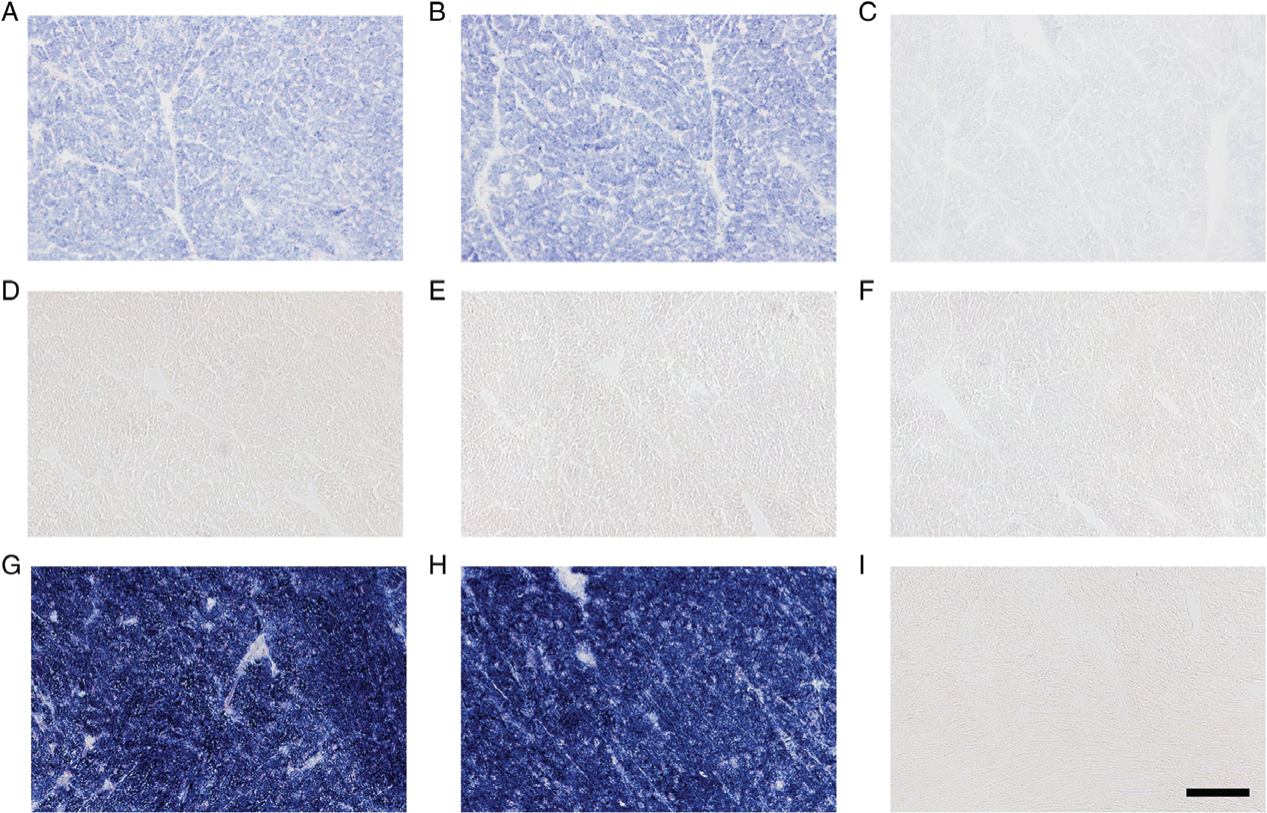
PMS engages both the activity of Complex II and the activity of Complex IV. (A) Succinic dehydrogenase activity is detected in wild‐type heart tissues using sodium succinate as a substrate and NBT as a final electron acceptor (n = 3). (B) Blocking COX activity with sodium azide does not prevent NBT reduction (n = 3). (C) Inhibiting the quinone‐binding site of Complex II with atpenin A5 fully blocks the transfer of electrons to NBT (500 nm, n = 3). Treatment of wild‐type heart tissues with a solution containing 0.2 mm PMS in addition to sodium succinate and NBT significantly represses the catalysis of blue formazan crystals for (D) 10 min, (E) 20 min or (F) 30 min incubation at 18 °C (n = 5). (G, H) Complete reduction of NBT is rescued by chemically blocking COX with (G) sodium azide (1 mm, 10 min, n = 15) or (H) potassium cyanide (1 mm, 10 min, n = 3). (I) Removing sodium succinate prevents formazan formation even in the presence of sodium azide (10 min, n = 3). Scale bar = 100 μm.

Using sodium succinate as a substrate and in the absence of PMS, NBT reduction led to visible accumulations of formazan – an indication of succinate dehydrogenase activity (Figure 1A). This activity was independent of Complex IV and no effect was observed when its oxidative activity was blocked with sodium azide (Figure 1B). However, formazan formation was strongly blocked by atpenin A5 (Figure 1C), a potent Complex II inhibitor 24.

Adding PMS has long been shown to improve the rate of formazan catalysis, but its use in tissue sections is known to be beneficial only with a COX inhibitor 21. We therefore speculated that PMS is capable of transferring electrons to Complex IV and that this reaction is favoured over its ability to reduce NBT. To confirm this, we incubated heart sections with PMS (0.2 mm), NBT (2 mm), and sodium succinate (130 mm) in the absence of any COX inhibitors. We observed that catalysis of formazan was suppressed, demonstrating the inability of PMS to reduce NBT in the presence of functional COX activity. NBT molecules remained in their oxidized form even after prolonged incubation (Figure 1D–F). As expected, NBT reduction was fully restored using sodium azide (1 mm) or potassium cyanide (1 mm), two COX inhibitors (Figure 1G, H). Removal of sodium succinate prevented this effect (Figure 1I).

To confirm the specificity of Complex IV in preventing the transfer of electrons from PMS to NBT, we attempted to restore NBT reduction with various electron transport chain inhibitors: Complex I (rotenone, 2 μm), Complex III (antimycin A, 1 μm and myxothiazol, 1 μm), and inhibited ATP synthesis (oligomycin A, 6 μm). None of the tested inhibitors could restore NBT reduction (supplementary material, Figure S1). In addition, the source of electron transfer from Complex II to PMS was confirmed using antimycin A, atpenin A5, and malonate (supplementary material, Figure S2). Our results indicate that once reduced by Complex II, PMS transfers electrons to COX or NBT, bypassing the classical respiratory chain electron pathway. Thus, in the presence of PMS, NBT reduction into formazan only depended on the respective activities of Complex II and COX.

**Figure 2:**
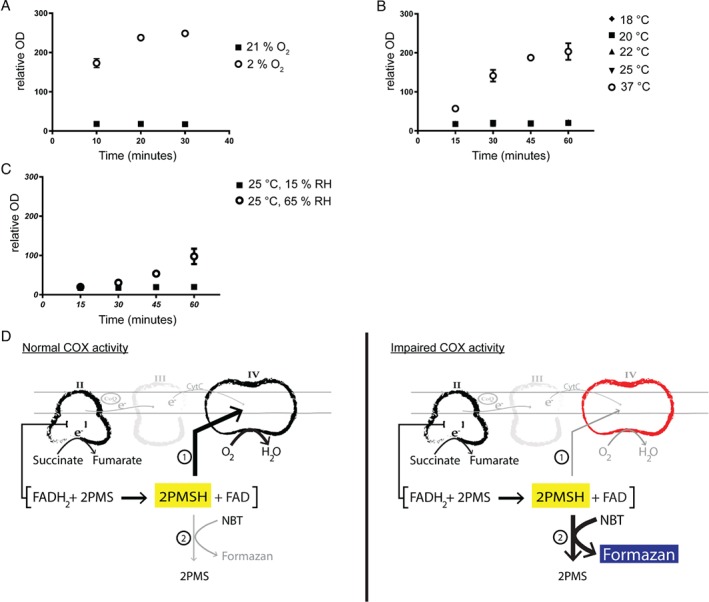
Competitive redox reactions. Wild‐type hearts from 12‐week‐old mice were used to test the stability of a negative signal. (A) Performing the NBTx assay in a hypoxic chamber (2% oxygen) leads to formazan deposition after 10 min at room temperature. (B) Slight variation in the temperature (between 18 and 25 °C) did not significantly increase formazan deposition, although high temperature (37 °C) for heart tissues causes gradual accumulation of formazan crystals. (C) High relative humidity levels (65%) increase formazan deposition in a healthy tissue and can influence the colourless background at a given temperature. N = at least 3 for each condition. (D) Proposed interaction scheme: PMS is reduced within Complex II and subsequently re‐oxidized by COX, leaving NBT in its colourless, oxidized form (marked as 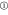). When the oxidative activity of Complex IV is either artificially blocked or deficient, PMS is efficiently passing electrons to NBT, which triggers the formation of blue formazan crystals (marked as 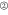).

As NBT reduction depends on the absence of COX activity and because oxygen is a substrate of COX and its concentration modulates COX catalytic activity and substrate affinity 25, we hypothesized that formazan formation could be influenced by oxygen pressure and availability. This possibility was examined with heart tissues of young (12 weeks old) wild‐type mice in a chamber containing 2% oxygen. Low‐oxygen conditions led to rapid accumulation of formazan pigments (Figure 2A). Likewise, elevated levels of formazan deposition were noted in young hearts incubated at high temperature, which we speculate increased the reaction rate and caused a more rapid depletion of dissolved oxygen in the incubation media (Figure 2B). High relative humidity also led to a slow deposition of formazan crystals over time, suggesting again that oxygen depletion is an important factor that influences the outcome of the NBTx assay (Figure 2C).

Our findings point to a strong interplay between PMS, Complex II, and Complex IV that is independent of the rest of the respiratory chain, in agreement with previous reports 17, 18, 19, 21. In light of these observations, we suggest a model where PMS interacts preferentially with Complex IV, hindering its ability to deliver electrons to NBT. In this scenario, NBT reduction serves as an indicator of the loss of COX activity and thus enables fast and sensitive detection of COX‐deficient cells (Figure 2D).

### COX deficiency in mouse models

**Figure 3:**
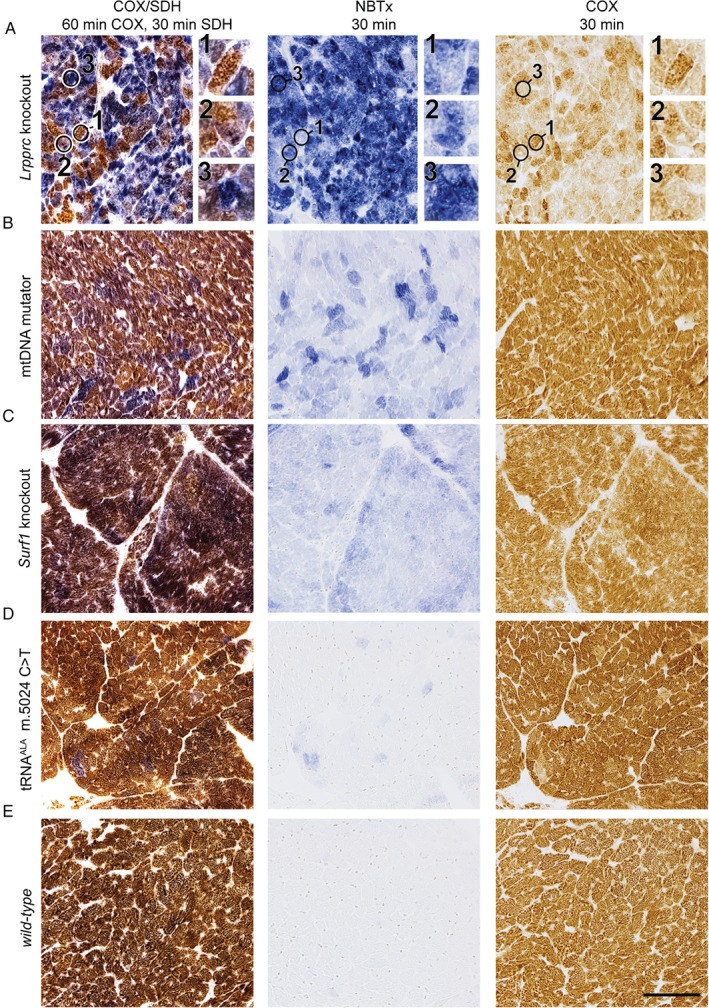
COX‐deficient cells in mouse heart tissues. Fresh‐frozen heart tissues from mouse models with COX deficiencies are shown here after COX/SDH, NBTx or COX alone. (A) Lrpprc knockout (n = 3); (B) mtDNA mutator (n = 3); (C) Surf1 knockout (n = 3); (D) mt‐tRNA ^ALA^ m.5024C > T (n = 5); (E) wild‐type (n = 5). The circles are identified areas with 1, low; 2, intermediate; or 3, high level COX deficiency. Scale bar = 100 μm.

Based on the biochemical interactions described above, we predicted that deficient cells within tissues of transgenic mice with known COX deficiency would be revealed by the deposition of formazan crystals. In order to verify this, we analysed four different transgenic mouse models previously characterised and presenting different levels of COX deficiency in the heart, namely: *Lrpprc* conditional knockout (*Lrpprc/CKMM‐cre*) 26, 27, *Surf1* knockout 28, 29, the *PolgA* ^*D257A*^ mtDNA mutator 30, and a mouse heteroplasmic for an mtDNA m.5024C > T mutation in the *tRNA* ^*ALA*^ gene (*mt‐tRNA* ^*ALA*^ mice) 31. *Lrpprc/CKMM‐cre* conditional knockout mice have an extensive loss of Complex IV in heart tissues, with enzyme activities decreased by 90% at 12 weeks of age 26. *Surf1* knockout mice are reported to suffer a loss between 30% and 50% of COX activity 27, 28, whereas mtDNA mutator mice lose around 25–30% 32. Mice harbouring the *mt‐tRNA* ^*ALA*^ mutation show only sparse COX impairment 31. The NBTx assay revealed that in these mouse models, qualitative levels of formazan crystal deposition correlated with the reported mitochondrial COX deficiency (Figure 3). The darkest blue pigments were seen in *Lrpprc* knockout heart sections, followed by *Surf1* knockout and the mtDNA mutator mouse, with the lowest amount being observed in mouse heart with the *mt‐tRNA* ^*ALA*^ mutation. The ease at which COX‐deficient cells are revealed was especially evident in the *Surf1* knockout model (Figure 3C). The relevance of this new approach is particularly striking when compared side‐by‐side with sequential COX/SDH or COX‐only histochemistry. The NBTx assay revealed even slight changes in COX activity, with cells clearly distinguishable with a light blue tint, whereas the same area in the COX/SDH serial section revealed a puzzling presentation of colours that varied from purple to brown or even greyish‐blue, which may lead to ambiguous interpretations and hinder proper quantification 12. Likewise, images of heart tissue reacted to DAB only show COX‐positive activity, which is a disadvantage when assessing for the presence of defective oxidative activity. For example, in the *Lrpprc* knockout heart tissue (Figure 3A), we identified three areas with as distinct intensity of formazan deposition (labelled 1 to 3) in the NBTx assay. The same areas in the COX/SDH section showed characteristic colour variation but the light blue seen in the NBTx section of area 1 was undetected when either the COX/SDH or the COX‐only histochemistry method was used. Furthermore, when COX deficiency was partial and equally distributed across the tissue (as seen in a *Surf1* knockout mouse), COX‐only histochemistry performed poorly for revealing COX‐deficient mitochondria (see Figure 3C). Overall, the newly developed NBTx assay successfully reveals COX‐deficient mitochondria in tissue sections and is more sensitive than previous methods.

### MtDNA heteroplasmy in heart tissues

**Figure 4:**
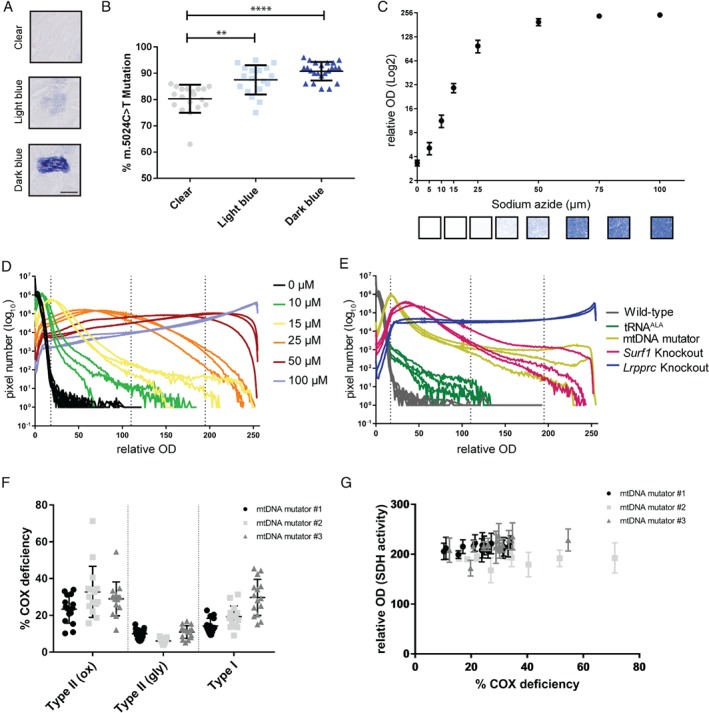
Blue pigment density correlates with heteroplasmy and COX deficiency levels. (A) The high contrast that ensued from the NBTx treatment of heart sections allows clear distinction of areas with low (clear), intermediate (light blue) or high (dark blue) formazan deposition. Scale bar = 20 μm. (B) Each area was collected separately with a laser dissecting microscope and analysed using pyrosequencing technology. Results of at least five pooled laser‐captured microdissections for each colour, from four different mice carrying heteroplasmic m.5024C > T mutation in the mt‐tRNA ^ALA^ gene. Single point values ±SD. Dunn's multiple comparisons test, one‐way ANOVA: clear versus light blue (**p ≤ 0.01) or clear versus dark blue (****p ≤ 0.0001). Light blue versus dark blue values are not statistically different. (C) Heart tissues were exposed to different concentrations of sodium azide (0, 5, 10, 15, 25, 50, 75, and 100 μm). Average blue intensity was calculated using Fiji software and plotted against each sodium azide concentration. Numbers represent mean ±SD from six images per animal (n = 3; 12‐week‐old males). Representative images for each azide concentration are shown under the graph. (D) Selected concentrations of sodium azide were plotted showing the total pixel number from six images against each ROD value. Dashed lines are arbitrary ranges for background, low, intermediate, and high deficiency, which we also transposed to tissues of the animal models. (E) Heart tissues of Lrpprc knockout, Surf1 knockout, mtDNA mutator, and mt‐tRNA ^ALA^ mice compared with age‐matched wild‐type tissue. Each line represents the results of replicate experiments for different animals. (F) COX deficiency measured in three different fibre types [type II (oxidative, ox), type II (glycolytic, gly), and type I] from the skeletal muscle of three mtDNA mutator mice. Each dot represents a single cell, with 15 cells per column, with mean and standard deviation shown. (G) Oxidative type II fibres were used to compare % COX deficiency with the value of SDH activity (relative OD; mean value of three replicates with standard deviation) obtained for each single cell.

Cellular heteroplasmy describes the presence of wild‐type and mutated copies of mtDNA molecules at a ratio that varies between organs and individual cells. The increase of this ratio towards the mutated variant is critical in the development of mtDNA‐induced mitochondrial disease or may be a risk factor in the development of other age‐related diseases 33. Using the NBTx assay, we were able to easily expose various levels of COX deficiency in heart tissues of mice with a point mutation in the *mt‐tRNA* ^*ALA*^ gene. Pups were assayed at weaning for their relative levels of the m.5024C > T mtDNA mutation using whole tissue quantitative pyrosequencing. Even mice with relatively high levels of the m.5024C > T mutation had low numbers of detectable dysfunctional cells within the tissue 31. Heart sections treated with the NBTx assay mixture revealed areas with low (clear), intermediate (light blue), or high (dark blue) formazan deposition (Figure 4A). We proceeded to isolate the different areas using laser capture microdissection with subsequent pyrosequencing to assay the relative m.5024C > T mutation level within the laser captured area. As expected, we observed a significant increase in the levels of the m.5024C > T mutation in dark or light blue areas, confirming a correlation between high relative levels of this pathogenic mutation and the presence of deficient COX activity (Figure 4B).

### Quantification of COX deficiency

A mosaic distribution of affected cells within a given tissue is a characteristic of many mitochondrial diseases. Quantifying this variation would help to decipher the correlation between cellular OXPHOS deficiencies and disease progression. To demonstrate the high sensitivity and reproducibility of the NBTx reaction, we proceeded to evaluate the degree of blue formazan crystals in wild‐type heart tissues exposed to increasing amounts of sodium azide. For this purpose, a simple and accessible workflow was developed using images captured with an upright microscope and subsequently analysed using Fiji, a free image processing software package (supplementary material, Table S1). Concentrations as low as 5 μm induced a small, detectable change in relative optical density (ROD) measurements, followed by a gradual increase which plateaued at about 100 μm. Linearity was detected in the range of 5–25 μm sodium azide (Figure 4C). Completely blocking mitochondrial activity with 1 mm sodium azide saturated the image with an average ROD of 252 ±1.5 (not shown).

The variation in cellular dysfunction within the tissue was lost when the average ROD was calculated rather than the single cell values. We reanalysed the images from the sodium azide‐treated heart tissues and extracted the total number of pixels for each intensity level. Figure 4D shows a distribution curve with an important distribution of values across the range, but interestingly each sodium azide concentration created a unique and consistent curve. Within a cell, variations in the density of formazan deposition between subcellular regions, as well as spaces between cells, might explain this distribution (see close‐up images in supplementary material, Figure S3). We arbitrarily assigned a range of blue optical densities which would represent low, intermediate, and high levels of COX deficiency, with the goal of establishing the distribution levels within our animal models. Plotting the number of pixels versus the blue ROD values for the four different animal models resulted in similar curves (Figure 4E). Moreover, the data for each animal were plotted individually to assess inter‐individual variability for each genotype.

The new NBTx assay accurately reveals COX deficiency with a very high sensitivity. The absence of brown pigments in functional mitochondria led to a clearer and consistent distinction of blue intensities. Our image analysis enables the extraction of valuable information such as COX deficiency levels – as an average ROD for the entire tissue or as a spectrum within the tissue (number of pixels per ROD); and variability within one specific genetic line [for example, we found that mtDNA mutator mice as well as *Surf1* knockout mice varied substantially between each animal, whereas *Lrpprc* knockout and *tRNA* ^*ALA*^ mice tended to be consistent (Figure 4E)].

In skeletal muscle tissue, we performed single cell analysis to look at the variability of the NBTx signal in fibre types which naturally vary in the levels of mitochondrial activity. Consecutive serial sections were used to distinguish fibre types with myosin ATPase reactions at pH 4.3 and 10, in addition to a section for histochemical assay of SDH activity. The intensity of formazan deposition, as measured in the SDH assay, was used to distinguish fibre types of similar mitochondrial activity (supplementary material, Figure S4 and Table S2). Three different types of fibres in mtDNA mutator mice hind limb muscle (gastrocnemius and soleus) – fibre type I, type II (oxidative), and type II (glycolytic) – were identified and used to make an evaluation of each fibre's characteristic COX impairment. We found no significant difference in SDH activity between wild‐type and mtDNA mutator animals (supplementary material, Figure S4E). Our results show that fibre types with greater oxidative activity [types II (oxidative) and I] show larger decreases in COX activity than the glycolytic fibres (Figure 4F). Also, measurements of individual cells from the same fibre type show a variation in COX impairment that does not correlate with the values of the measured SDH activity (Figure 4G).

### NBTx assay of other tissues

**Figure 5:**
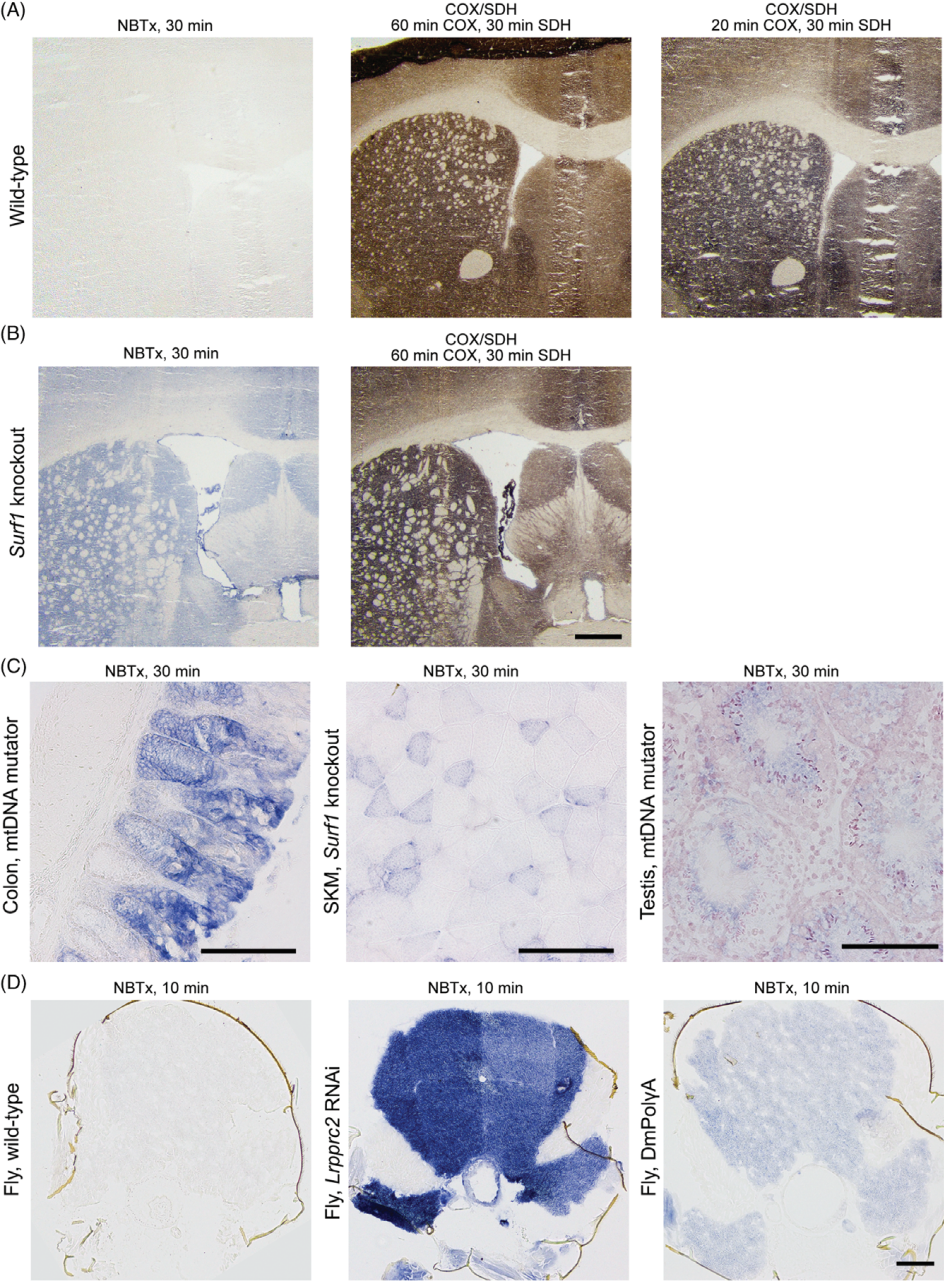
NBTx assay in a variety of tissues. (A) Brain sections of wild‐type mice are shown stained either with the NBTx or the COX/SDH method. (B) Consecutive section of brain Surf1 knockout mice (n = 3). Scale bar = 500 μm. (C) Colons and testes of mtDNA mutator mice as well as skeletal muscles (SKM) of Surf1 knockout mice (n = 3). Scale bar = 100 μm. (D) Skeletal muscles from the thorax of Drosophila melanogaster stained for 10 min at 18 °C. Representative images of five flies per genotype. Scale bar = 100 μm.

The suitability of the NBTx assay in other mouse organs was investigated. We were particularly interested in looking at brain sections, as this organ had proven to require extensive and laborious optimization with traditional COX/SDH 11, 12. NBTx assays on brain sections of *Surf1* knockout mice revealed an easily observable deposition of blue pigments across the tissue (Figure 5A, B), whereas we found the serial sections of control and mutant brain sections after a COX/SDH reaction difficult to interpret (Figure 5A, B, second panel). Similar to the heart, the resulting colour was similar to unoptimized conditions, where DAB incubation was insufficient to block SDH activity in normal brain controls (Figure 5A, 20 min COX, third panel). Within these COX/SDH sections, it appeared as though brown deposits within functional mitochondria were mixed with blue deposits, rendering the coloration of *Surf1* knockout brain sections difficult to interpret. In contrast, detection of COX‐deficient cells against the background of COX‐functional cells that lack any coloration provided excellent contrast with the NBTx assay.

We proceeded with the evaluation of a few other mouse organs that are commonly used in our laboratories to test how other tissues respond to the NBTx assay. Deficient COX activity was easily revealed in colonic crypts and skeletal muscles (Figure 5C, first and second panels). Furthermore, small developing sperm cells in the testis were also successfully detected with the NBTx method (Figure 5C, third panel). In the latter, strong pink discoloration was seen in addition to the blue crystals and was interpreted as a half‐reduced intermediate which is partly washed away in ethanol 34, 35.

### NBTx assay to study Drosophila melanogaster

The fruit fly *Drosophila melanogaster* is becoming more commonly used as an alternative animal model for mitochondrial research, yet histochemical techniques are lacking for these models 36. The fly *Lrpprc2* gene is one of two homologs of the mouse *Lrpprc* gene, which is involved in coordinating mitochondrial gene translation, and *Lrpprc2* knockout or knockdown leads to a drastic loss of respiratory chain complexes 37. The DmPolγA flies are flies trans‐complementing two deleterious mitochondrial polymerase gamma mutant alleles (the exo‐deficient *PolgA* ^*D257A*^ and the processivity‐impairing *PolgA* ^*H1134A*^ alleles), which present no obvious pathogenic phenotypes 38. However, using the NBTx assay, we were able to observe mild deficiency in COX activity. The high Complex IV content in the fly thoracic muscles required a short incubation time of only 10 min at 18 °C to produce strong formazan deposition in the *Lrpprc2* RNAi fly. DmPolγA muscles revealed mild formazan deposition, whereas wild‐type healthy muscles remained colourless under these conditions (Figure 5D).

### NBTx assay in the diagnosis of mitochondrial diseases

**Figure 6:**
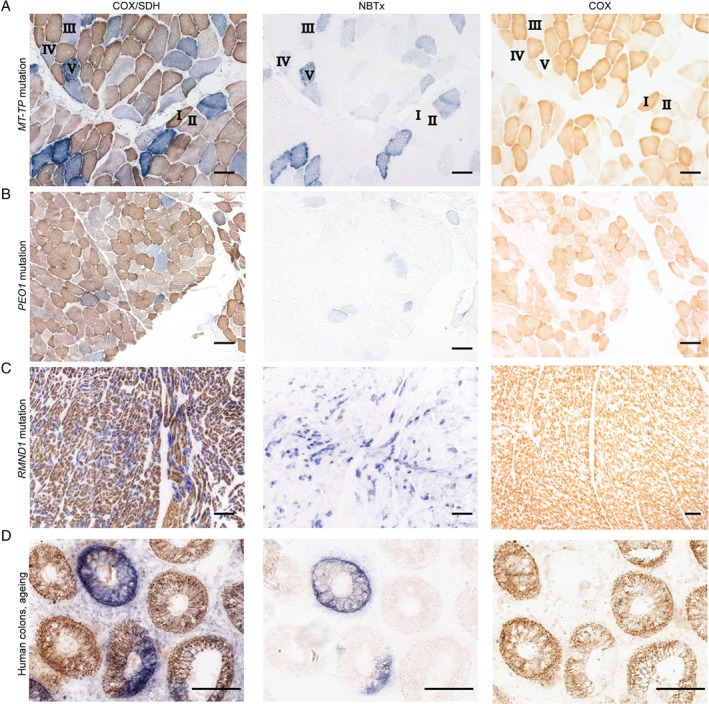
Human tissues analysed using the COX/SDH, NBTx or COX histochemical methods. (A) Skeletal muscle tissue with a novel m.16015 T > C MTTP mutation. Roman numerals indicate individual cells with (I) no COX deficiency, high COX activity; (II) no COX deficiency, low COX activity; (III) mild COX deficiency, low SDH activity levels; (IV) mild COX deficiency, mild SDH activity levels; and (V) strong COX deficiency, high SDH activity levels. (B) Section of skeletal muscle from a patient with identified nuclear gene p.(Arg371Trp) TWNK (formerly PEO1) mutation. (C) Human heart tissue, p.Asn238Ser;c.829_830 + 2her_delGAGT RMND1 mutations. (D) Sections of colonic tissue from a patient with no known mitochondrial disease. COX‐deficient colonic crypts are detected in elderly patients (n = 2; 78 and 72 years old). Scale bar = 100 μm.

The sequential COX/SDH histochemical assay is universally applied in pathology laboratories as part of the diagnostic work‐up of human tissue, including muscle biopsies, to identify COX‐deficient cells 39. To test the applicability of the NBTx method in human diagnostics, we compared serial sections of NBTx images against sequential COX/SDH and COX‐only reactions for a variety of human biopsy samples (Figure 6).

Seven distinct genotypes were used to evaluate human skeletal muscles as well as seven samples of heart tissues (supplementary material, Figures S5 and S6). We found that regardless of the genetic defect (mtDNA mutations, Figure 6A or nuclear gene mutations, Figure 6B, C), the NBTx assay successfully exposed the full range of COX‐deficient cells, as predicted from the COX/SDH sections, with the advantage that low and intermediate levels of dysfunction were distinguished with a quantifiable variation in blue intensity. In contrast to the classical COX/SDH reaction, the NBTx assay does not identify cells with functional mitochondria, regardless of the COX activity level (Figure 6A, marked I and II), but, as seen in the mouse, reveals dysfunctional COX activity with the accumulation of a blue formazan pigment of varying intensity (Figure 6A, marked III, IV, and V). Thus, NBTx successfully detected COX‐deficient cells in all three types of tissue in patterns consistent with the COX/SDH method. These data show that the NBTx assay is suitable for human diagnostic work, revealing high‐contrast sections that allow for quantification and rapid diagnosis.

## Discussion

Accurately quantifying COX impairment at the cellular level will contribute to our understanding of the role of respiratory chain defects in mitochondrial disease processes and the implication of mitochondrial dysfunction in age‐related diseases. The currently available methods for cell‐specific detection, such as COX‐only histochemistry, immunolabelling of mitochondrial complex subunits or the sequential COX/SDH assay, either involve many steps or are limited to the detection of high levels of COX deficiency, failing to clearly expose low or intermediate levels. We have shown that deficiency in cytochrome *c* oxidase activity can be detected with the simple reduction of NBT. We have demonstrated here that the NBTx method exploits tightly competing biochemical interactions between PMS, NBT, and Complex IV to evaluate the integrity of COX activity in an enzyme histochemical assay, eliminating the need for DAB. The presence of DAB has been reported to interfere with certain assays, such as real‐time qPCR 12, and therefore the capacity to reveal COX‐deficient cells without DAB is advantageous in many ways. The assay generates images with a high contrast between COX‐deficient and COX‐competent cells, and not only reduces the overall cost but is also quick and allows clear identification of cells harbouring even mildly COX‐deficient mitochondria. This single‐colour reaction has the added advantage that simple automation and quantitative analysis are possible, without having to rely on comparing the activity levels between samples (as is done with COX histochemical methods or immunohistochemistry).

The one‐step NBTx assay eliminates the confounding results observed with the sequential COX/SDH reaction of intermediate COX deficiency 12. Unarguably, the strong contrast of blue COX‐deficient cells over a clear background of fully functional cells in the NBTx assay offers an improvement over previous methods. Our results on brain sections of *Surf1* constitutive KO mice illustrate how intermediate levels of COX activity are easily identified. Beyond primary mitochondrial disease, loss of mitochondrial activity in the brain is a domain of investigation in normal ageing processes or age‐related diseases such as Alzheimer's or Parkinson's disease 40, 41, 42. Reduction of COX activity in aged brain tissue has been reported in humans, rodents, and monkeys using COX cytochemical or immunohistochemical methods 43, 44, 45, 46. Analysis of DAB accumulation or detection of immunolabelled Complex IV subunits is challenging because both require imaging at very precise brain areas and comparing those areas between samples. This limits the field of view to precisely where investigators make their measurements and given the heterogeneous distribution of mitochondrial activity in the brain, assessment of loss of enzyme activity in the nervous system is difficult.

Based on the successful identification of COX deficiency in human tissues of a wide range of genetic backgrounds, we set the basis for establishing the NBTx assay within the diagnostic workflow. In addition, we have shown here the potential for the acquisition of quantitative data, which will support research on mitochondrial disease mechanisms and also in the evaluation of therapeutic approaches. We have also demonstrated that the method is easily re‐optimized for a variety of mouse or human tissues and smaller model organisms such as the fly *Drosophila melanogaster*, and we anticipate that the advantages of this method will benefit many in the field of mitochondrial research.

## Author contributions statement

MLS developed the NBTx assay, conceived and designed the study, carried out experiments, analysed data, and prepared the manuscript. AM and JBS conceived experiments, analysed data, and supervised the writing of the manuscript. LCG and RWT provided human tissues and supervised experiments in Newcastle upon Tyne. All authors had final approval of the submitted and published versions.

**SUPPLEMENTARY MATERIAL ONLINE**

**Supplementary materials and methods**

**Supplementary figure legends**

**Figure S1.** Inhibiting mitochondrial complexes other than Complex IV does not re‐establish the reduction of NBT

**Figure S2.** Site of electron transfer from Complex II to PMS

**Figure S3.** Distribution of formazan crystals

**Figure S4.** Quantification of skeletal muscle fibre type

**Figure S5.** Skeletal muscle sections from patients with various genetic backgrounds

**Figure S6.** Heart tissues from patients with various genetic backgrounds

**Table S1.** Fiji macro for NBTx quantitative analysis

**Table S2.** Skeletal muscle raw data

## Supporting information


**Supplementary materials and methods**

**Supplementary figure legends**

**Figure S1.** Inhibiting mitochondrial complexes other than Complex IV does not re‐establish the reduction of NBT. Heart sections from wild‐type mice were incubated for 10 min in NBTx solution containing the following inhibitors: (A) rotenone (2 μm), (B) antimycin A (1 μm), (C) myxothiazol (1 μm), and (D) oligomycin (6 μm). Representative images of three experiments. Scale bar = 100 μm.

**Figure S2.** Site of electron transfer from Complex II to PMS. (A) NBTx + sodium azide (1 mm); (B) NBTx + sodium azide and antimycin A (1 μm); (C) NBTx + sodium azide and atpenin A5 (5 μm); (D) NBTx + sodium azide and malonate (6 mm). Representative images of three experiments. Scale bar = 100 μm.

**Figure S3.** Distribution of formazan crystals. (A) Images of a section of heart tissue exposed to 75 μm sodium azide and the NBTx solution. Arrows show unstained areas of connective tissues or holes in the tissue. Image inset shows the non‐homogenous intensity of formazan deposition within the cytoplasm.

**Figure S4.** Quantification of skeletal muscle fibre types. Consecutive sections of the gastrocnemius and soleus muscles of mtDNA mutator mice were treated with (A, E) myosin ATPase at pH 4.3, (B, F) myosin ATPase at pH 10, (C, G) SDH assay, and (D, H) NBTx assay. SDH and NBTx reactions were run simultaneously at 18°C for 30 min. (I) Relative optical density (ROD) depicting SDH activity in the skeletal muscle. Individual cells from type II (oxidative, ox or glycolytic, gly) and type I fibres were selected in wild‐type and mtDNA mutator mice. Mean relative OD ± SD (n = 3). Scale bar = 100 μm.

**Figure S5.** Skeletal muscle sections from patients with various genetic backgrounds. Tissue was cut at 10 μm and consecutive slides were used for comparing COX/SDH with the new NBTx method. Scale bar = 100 μm.

**Figure S6.** Heart tissues from patients with various genetic backgrounds. Tissue was cut at 10 μm and consecutive slides were used for comparing COX/SDH with the new NBTx method. Scale bar = 100 μm**.**

**Table S1**. Fiji Macro for NBTx Quantitative Analysis

**TableS 2** Wild‐Type: SDH activity
