# Supplementary material for: A novel histochemistry assay to assess and quantify focal cytochrome c oxidase deficiency
Source: J Pathol. 2018 May 14;245(3):311–23. doi: 10.1002/path.5084 (PMC6032845; doi:10.1002/path.5084)
Supplement: Supplementary file 1 — Supplementary materials and methods [file PATH-245-311-s001.pdf]

Supplementary materials and methods

Reference numbers refer to the main text reference list

Mouse tissues

All the mouse lines used in all the experiments were maintained on a C57Bl/6NCrl nuclear background (Charles River Laboratories, Erkrath, Germany; strain code 027). *Lrpprc* conditional knockouts (MGI: 5438915) and *Surf1* knockout mice (MGI: 2651426) were kindly provided by the group of Nils-Göran Larsson, Department of Mitochondrial Biology, Max Planck Institute for Biology of Ageing, Cologne. mtDNA mutator mice (MGI: 3046825) were F1 animals born to true C57Bl/6NCrl females from the P generation to minimize mtDNA mutagenesis [31]. The *mt-tRNA^{ALA}* m.5024C>T mice (MGI: 5902095) are maintained by female transmission of the mutated mtDNA in crosses to C57Bl/6NCrl males.

All animal work was performed in accordance with recommendations and guidelines of the Federation of European Laboratory Animal Science Associations (FELASA). All experiments were approved and permitted by the Landesamt für Natur, Umwelt und Verbraucherschutz Nordrhein-Westfalen in accordance with German and European Union regulations (Permits 84-02.042015.A103 and 84-02.05.50.15.004).

Human tissues

Human skeletal muscle (vastus lateralis) and cardiac tissue were obtained through the NHS Highly Specialised Services located within the Wellcome Centre for Mitochondrial Research at Newcastle University. All samples were obtained and used with informed consent. This study was approved and performed under the ethical guidelines issued by the Newcastle and North

Tyneside Local Research Ethics Committees (reference 09/H0906/75) and complied with the Declaration of Helsinki. Human colon tissue was obtained from patients with colorectal tumours undergoing surgical resection. Normal mucosa was taken from at least 20 cm from the resection margin. Informed written consent was obtained prior to surgery and samples were coded to maintain confidentiality. This project was approved by the Joint Ethics Committee of Newcastle and North Tyneside Health Authority (REC ref. No 2001/188). All tissues were cut at 7 µm and consecutive sections were used to compare COX/SDH (40 min COX, 40 min SDH at 37°C), NBTx (20 min at 37°C), and COX-only (skeletal muscle and heart sections – 40 min; colon sections – 30 min at 37°C).

Fruit flies, *Drosophila melanogaster*

Fly models were kindly provided by the group of Nils-Göran Larsson, Department of Mitochondrial Biology, Cologne, Germany [see refs 37 and 38 for maintenance and engineering of *Lrpprc2* RNAi and DmPolγA (compound heterozygous tam^{D263A}/tam^{H1038A}), respectively]. Flies were quickly frozen in liquid nitrogen and subsequently glued onto a cryostat mounting disk with fixing gel (Tissue-Tek® O.C.T. compound). Sections of the thorax muscles were cut at 10 µm and mounted on microscope slides. NBTx assays were performed at 18°C for 10 min.

Hypoxic conditions

Heart tissues were cut and sections placed on microscope slides as for the standard NBTx experiments. PBS buffer was placed with lid open inside an oxygen controlling chamber system (COY Laboratory Products, Grass Lake, MI, USA) 30 min before the start of the experiment. Chamber parameters were set for 2.0% oxygen and 5.0% CO_2. Temperature and humidity inside

the chamber were measured around 24°C and 22% relative humidity. Microscope slides with 7-µm-thick sections of mouse heart tissue were placed inside the chamber. PBS buffer was added to the slides and incubated for 10 min, followed by the usual NBTx buffer for 10, 20 or 30 min. After the incubation time, slides were taken out of the chamber and transferred into water for 5 min, and dehydrated in ethanol and xylene before mounting with coverslips.

COX/SDH assay

The protocol used here was a standard protocol utilized across diagnostic histopathology laboratories [11].

Succinate dehydrogenase (SDH) activity

SDH activity was measured by removing PMS from the NBTx staining solution. Without the presence of PMS as an intermediate electron carrier, the catalysis of NBT into formazan crystals is weak but independent of the activity of Complex IV. Alternatively, to mimic 100% COX deficiency in tissues, SDH activity was also assessed with 1 mM sodium azide in the NBTx solution.

DAB histochemistry

3,3'-Diaminobenzidine tetrahydrochloride (DAB) was dissolved in purified water followed by an equal volume of 0.2 M phosphate buffer, pH 7.0, for a final DAB concentration of 5 mM. Prior to staining, cytochrome *c* (100 µM) and catalase (0.2 mg/ml) were added.

Laser microdissection and quantitative pyrosequencing

Heart tissues were cut at 12 µm thickness, collected onto PEN-membrane slides (Leica Microsystems GmbH, Wetzlar, Hesse, Germany), and stained for 30 min using the NBTx assay. Areas stained blue or areas with no coloration were then laser micro-dissected (LMD7000, Leica) and collected into sterile 0.5 ml centrifuge tubes. The collected sections were lysed in 10 µl of lysis buffer [50 mM Tris–HCl (pH 8.5), 1% Tween-20, 20 mg/ml proteinase K] followed by a 2 h incubation at 55°C. Proteinase K was then denatured by a 10 min incubation at 95°C. Pyrosequencing analysis of the m.5024C>T mutation was performed using a PyroMark Q24 pyrosequencer and the design software v2.0 (Qiagen GmbH, Hilden, Germany). A single PCR reaction was used to amplify a 178 bp PCR fragment spanning the m.5024 mutation site, using a biotinylated forward primer and a non-biotinylated reverse primer (forward primer: 5'-biotin-TTCCACCCTAGCTATCATAAGC; reverse primer: GTAGGTTTAATTCCTGCCAATCT). PCR products were combined with dH_2O, PyroMark binding buffer (Qiagen), and 1 µl Streptavidin Sepharose™ high performance beads (GE Healthcare, Little Chalfont, UK), and purified and denatured using a Pyromark Q24 vacuum workstation. Sequencing was carried out using PyroMark Gold Q24 Reagents according to the manufacturer’s directions, using the sequencing primer (TGTAGGATGAAGTCTTACA). For calibration curves, mixtures of synthetic biotin-labelled oligos were added to wild-type and mutant sequences.

Statistical analyses

Pyro sequencing data were analysed using GraphPad Prism 7 software. A one-way ANOVA with Dunn’s multiple comparisons test was used to establish statistical significance between the

percentage of m.5024C>T mutations in clear versus light blue areas, white versus dark blue areas, and light blue versus dark blue areas.

Image acquisition, processing, and analysis

Images were obtained using an Eclipse Ci microscope (Nikon Instruments, Duesseldorf, Germany) and processed with Fiji (open-source software [47]). The following steps were written in a single macro command: white balancing, colour deconvolution, and threshold [48], followed by total image analysis of the area, mean intensity, and standard deviation values. In addition, processed images were stacked and the total pixel number for each intensity value (0–255) was analysed with the histogram command. Further details about the macro are provided in the supplementary material, Table S1. The macro is written to process many images at once, making it easier to analyse several images. When prompted by the macro, select a folder containing the images.

Quantitative analysis of skeletal muscle

Skeletal muscles of mouse hind limbs (gastrocnemius and soleus) were cut in serial sections at 10 μm for NBTx and SDH assays, and at 12 μm for myosin ATPase reactions. Images processed with the macro were loaded to ImageJ again and single fibres were selected and analysed individually. Excel and GraphPad Prism 7 were used to compare maximal formazan catalysis (SDH assay) with the blue intensity generated with the NBTx reaction (mean relative OD). Single cells were identified in three consecutive sections placed side-by-side on one slide. Values were normalized to the mean intensity of a large area of each slide to account for variability (see also supplementary material, Table S2, for raw data).
