# Supplementary material for: A novel histochemistry assay to assess and quantify focal cytochrome c oxidase deficiency
Source: J Pathol. 2018 May 14;245(3):311–23. doi: 10.1002/path.5084 (PMC6032845; doi:10.1002/path.5084)
Supplement: Supplementary file 2 — Supplementary figure legends [file PATH-245-311-s010.pdf]

Supplementary figure legends

Figure S1. Inhibiting mitochondrial complexes other than Complex IV does not re-establish the reduction of NBT. Heart sections from wild-type mice were incubated for 10 min in NBTx solution containing the following inhibitors: (A) rotenone (2 µM), (B) antimycin A (1 µM), (C) myxothiazol (1 µM), and (D) oligomycin (6 µM). Representative images of three experiments. Scale bar = 100 µm.

Figure S2. Site of electron transfer from Complex II to PMS. (A) NBTx + sodium azide (1 mM); (B) NBTx + sodium azide and antimycin A (1 µM); (C) NBTx + sodium azide and atpenin A5 (5 µM); (D) NBTx + sodium azide and malonate (6 mM). Representative images of three experiments. Scale bar = 100 µm.

Figure S3. Distribution of formazan crystals. (A) Images of a section of heart tissue exposed to 75 µM sodium azide and the NBTx solution. Arrows show unstained areas of connective tissues or holes in the tissue. Image inset shows the non-homogeneous intensity of formazan deposition within the cytoplasm.

Figure S4. Quantification of skeletal muscle fibre types. Consecutive sections of the gastrocnemius and soleus muscles of mtDNA mutator mice were treated with (A, E) myosin ATPase at pH 4.3, (B, F) myosin ATPase at pH 10, (C, G) SDH assay, and (D, H) NBTx assay. SDH and NBTx reactions were run simultaneously at 18°C for 30 min. (I) Relative optical density (ROD) depicting SDH activity in the skeletal muscle. Individual cells from type II

(oxidative, ox or glycolytic, gly) and type I fibres were selected in wild-type and mtDNA mutator mice. Mean relative OD ± SD (n = 3). Scale bar = 100 µm.

Figure S5. Skeletal muscle sections from patients with various genetic backgrounds. Tissue was cut at 10 µm and consecutive slides were used for comparing COX/SDH with the new NBTx method. Scale bar = 100 µm.

Figure S6. Heart tissues from patients with various genetic backgrounds. Tissue was cut at 10 µm and consecutive slides were used for comparing COX/SDH with the new NBTx method. Scale bar = 100 µm**.**
