# Supplementary material for: A novel histochemistry assay to assess and quantify focal cytochrome c oxidase deficiency
Source: J Pathol. 2018 May 14;245(3):311–23. doi: 10.1002/path.5084 (PMC6032845; doi:10.1002/path.5084)
Supplement: Supplementary file 9 — Table S1. Fiji Macro for NBTx Quantitative Analysis [file PATH-245-311-s008.pdf]

Fiji Macro for NBTx Quantitative Analysis

Before

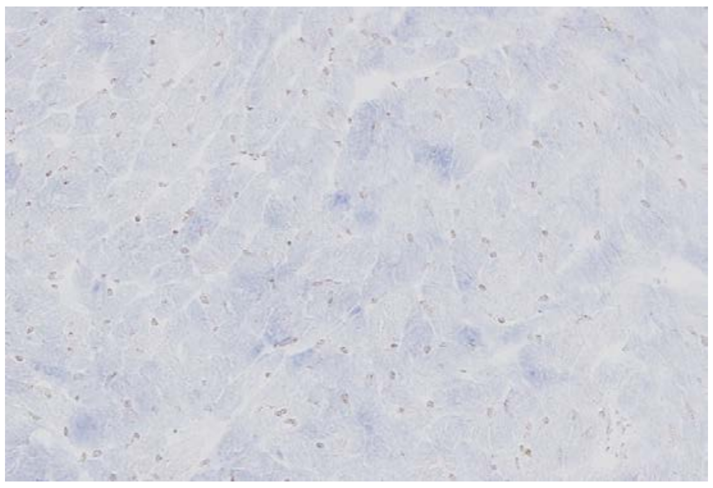

After

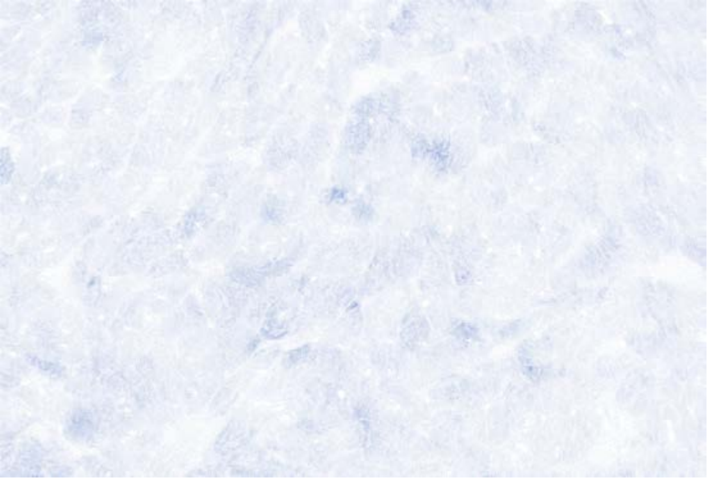

```
optionalManualRoiSelection=0;//if 1 manual ROI selection is offered for each image, if 0 autothresholding is applied without callback
directory=getDirectory("Choose a Directory");
filelist=getFileList(directory);
filelist=cleanFilelist(filelist);
resultpath=directory+File.separator+"ImageJ";
origFile="";
if(!File.exists(resultpath)){
	File.makeDirectory(resultpath);
}
run("Clear Results");
run("Set Measurements...", "area mean standard min median area_fraction redirect=None decimal=3");
totalArea=newArray(filelist.length);
for(i=0;i<filelist.length;i++){
	open(filelist[i]);
	origImg=getImageID;
	origFile=File.nameWithoutExtension;
	deconvFile=resultpath+File.separator+origFile+"_"+i+".tif";
	selectImage(origImg);
	//TODO: Implement white balancing
	//TODO: Implement interactive component vector creation
	run("Colour Deconvolution", "vectors=[User values] [r1]=107 [g1]=86 [b1]=37 [r2]=12 [g2]=17 [b2]=14 [r3]=10 [g3]=10 [b3]=10");
	//selectWindow("02_1345_NBTx15min_22DC_080616_001.tif-(Colour_3)");
	close("*Colour_2*");
	//selectWindow("02_1345_NBTx15min_22DC_080616_001.tif-(Colour_2)");
	close("*Colour_3*");
	//selectWindow("02_1345_NBTx15min_22DC_080616_001.tif-(Colour_1)");
	deconvImg=getImageID;
	run("Invert");
	run("Invert LUT");
	run("Duplicate...", " ");
	maskImg=getImageID;
	setAutoThreshold("Huang");
	setOption("BlackBackground", true);
	run("Convert to Mask");
	run("Create Selection");
	if(optionalManualRoiSelection){
		discardAutothreshold=getBoolean("Would you like to change the selected ROI?");
		if(discardAutothreshold){
			close;
			selectImage(deconvImg);
			run("Duplicate...", " ");
			maskImg=getImageID;

			//Prompt until user selected ROI
			setSelection=-1;
			while(setSelection<0){
				waitForUser("Create user defined selection");
				setSelection=selectionType();
			}
		}
	}
	roiManager("Add");
	selectImage(deconvImg);
	roiManager("Select", i);
```

```
		run("Measure");
		selectImage(deconvImg);
		run("Select All");
		getStatistics(area);//assumes constant image size!
		totalArea[i]=area;
		save(deconvFile);
		roiManager("Save", resultpath+File.separator+"RoiSet.zip");
		close("*");
}
saveAs("Results", resultpath+File.separator+"Values.csv");
roiManager("reset");
plottitle=origFile;

xlabel="Image number";
ylabel="Stain Intensity [a.u.]";
thrshAreaValues=newArray(nResults);
thrshMeanValues=newArray(nResults);
thrshStdDevValues=newArray(nResults);

for(i=0;i<nResults;i++){
		thrshAreaValues[i]=getResult("Area", i);
		thrshMeanValues[i]=getResult("Mean", i);
		thrshStdDevValues[i]=getResult("StdDev", i);
}
yValues=thrshMeanValues;

Plot.create(plottitle, xlabel, ylabel);
//Plot.setLimits(0, 5, 0, 3);
Plot.setLineWidth(5);
Plot.setColor("darkGray");
Plot.add("line", thrshMeanValues);
Plot.add("error bars", thrshStdDevValues);


Plot.setColor("lightGray");
for(i=0;i<nResults;i++){
		x=i*(1/(nResults-1));
		areaPercentage=round((thrshAreaValues[i]/totalArea[i])*100);
		Plot.addText(areaPercentage+"%", x, 0.12);
		row=nResults-(nResults-i);
		setResult("%Area",row,areaPercentage);
}


Plot.show();
saveAs("png",resultpath+File.separator+"Plot.png");

function cleanFilelist(filelist){
		origLength=filelist.length;
		cleanLength=origLength;
		for(i=0;i<origLength;i++){
				if(!endsWith(filelist[i],".tif")){
						cleanLength=cleanLength-1;
						filelist[i]="-";
				}
		}
		if(cleanLength==origLength) {
				return filelist;
		}

		cleanList=newArray(cleanLength);
		idx=0;
		for(i=0;i<origLength;i++){
				if(filelist[i]!="-"){
						cleanList[idx]=filelist[i];
						idx=idx+1;
				}
		}
		return cleanList;
}
```
