# Supplementary material for: A novel histochemistry assay to assess and quantify focal cytochrome c oxidase deficiency
Source: J Pathol. 2018 May 14;245(3):311–23. doi: 10.1002/path.5084 (PMC6032845; doi:10.1002/path.5084)
Supplement: Supplementary file 10 — TableS 2 Wild‐Type: SDH activity [file PATH-245-311-s009.pdf]

Wild-Type: SDH activity

Adjustment Factor:

	Sneu26	AF	SNeu27	AF	SNeu28	
S1	120.136	1.018119	165.394	1.01	91.765	0.96
S2	124.851	1.058077	165.395	1.01	99.677	1.04
S3	109.007	0.923804	162.904	0.99		
	117.998		164.5643		95.721	

Type I

Cell #	Sneu26								Sneu27							
	S1S1	Adjusted	S1S2	Adjusted	S1S3	Adjusted	Average	SD	S1S1	Adjusted	S1S2	Adjusted	S1S3	Adjusted	Average	SD
1	154.645	151.6127	163.767	154.4972	131.438	142.8674	149.6591	6.056028	180.801	179.0109	183.133	181.3198	166.148	167.8263	176.0523	7.216908
2	135.693	133.0324	149.621	141.1519	116.309	126.4228	133.5357	7.377419	154.034	152.5089	145.778	144.3347	144.123	145.5788	147.4741	4.40441
3	162.047	158.8696	166.867	157.4217	121.977	132.5837	149.625	14.77595	180.426	178.6396	172.528	170.8198	164.725	166.3889	171.9494	6.202987
4	170.194	166.8569	178.562	168.4547	147.692	160.5348	165.2821	4.188221	178.028	176.2653	165.173	163.5376	166.523	168.2051	169.336	6.438791
5	139.565	136.8284	152.256	143.6377	132.912	144.4696	141.6452	4.192165	155.136	153.6	151.702	150.2	146.012	147.4869	150.429	3.06299
6	142.232	139.4431	137.192	129.4264	127.283	138.3511	135.7402	5.495105	183.69	181.8713	208.585	206.5198	184.71	186.5758	191.6556	13.08591
7	133.094	130.4843	132.479	124.9802	128.146	139.2891	131.5845	7.21764	167.032	165.3782	177.197	175.4426	167.043	168.7303	169.8504	5.124814
8	153.324	150.3176	145.522	137.2849	126.416	137.4087	141.6704	7.488977	188.186	186.3228	199.381	197.4069	177.68	179.4747	187.7348	9.049099
9	151.708	148.7333	142.833	134.7481	135.874	147.6891	143.7235	7.79045	173.069	171.3554	172.178	170.4733	173.071	174.8192	172.216	2.297203
10	157.244	154.1608	161.165	152.0425	144.307	156.8554	154.3529	2.412235	183.294	181.4792	178.408	176.6416	161.174	162.802	173.6409	9.693412
11	146.665	143.7892	161.312	152.1811	143.001	155.4359	150.4687	6.009189	157.003	155.4485	157.237	155.6802	163.242	164.8909	158.6732	5.385934
12	142.705	139.9069	137.051	129.2934	123.39	134.1196	134.4399	5.313981	170.703	169.0129	174.151	172.4267	161.978	163.6141	168.3512	4.443394

Cell #	Sneu28							
	S1S1	Adjusted	S1S2	Adjusted	S1S3	Adjusted	Average	SD
1	182.998	190.6229	162.614	156.3596			173.4913	24.22781
2	149.796	156.0375	122.238	117.5365			136.787	27.22429
3	184.765	192.4635	139.227	133.8721			163.1678	41.43039
4	170.665	177.776	127.563	122.6567			150.2164	38.97524
5	124.73	129.9271	97.766	94.00577			111.9664	25.4002
6	138.201	143.9594	84.969	81.70096			112.8302	44.02335
7	144.194	150.2021	107.473	103.3394			126.7708	33.1369
8	144.331	150.3448	116.672	112.1846			131.2647	26.98332
9	154.753	161.201	131.326	126.275			143.738	24.69644
10	134.854	140.4729	113.211	108.8567			124.6648	22.35602
11	161.851	168.5948	137.592	132.3			150.4474	25.66429
12	146.134	152.2229	125.628	120.7962			136.5095	22.22208

Type II (gly)

Cell #	Sneu26								Sneu27							
	S1S1	Adjusted	S1S2	Adjusted	S1S3	Adjusted	Average	SD	S1S1	Adjusted	S1S2	Adjusted	S1S3	Adjusted	Average	SD
1	58.955	57.79902	50.801	47.92547	39.733	43.18804	49.63751	7.454426	105.658	104.6119	123.426	122.204	120.387	121.603	116.1396	9.987839
2	69.371	68.01078	73.205	69.06132	50.165	54.52717	63.86643	8.105068	88.426	87.5505	98.022	97.05149	90.183	91.09394	91.89864	4.801339
3	56.922	55.80588	65.393	61.69151	44.588	48.46522	55.32087	6.626472	88	87.12871	77.813	77.04257	84.603	85.45758	83.20962	5.405786
4	86.611	84.91275	80.227	75.68585	59.14	64.28261	74.9604	10.33418	74.94	74.19802	77.177	76.41287	90.041	90.95051	80.52047	9.100312
5	64.004	62.74902	53.913	50.86132	44.304	48.15652	53.92229	7.762885	87.493	86.62673	88.142	87.26931	84.275	85.12626	86.34077	1.099769
6	75.812	74.32549	78.487	74.04434	59.214	64.36304	70.91096	5.672402	128.367	127.096	123.659	122.4347	102.576	103.6121	117.7143	12.43323
7	84.947	83.28137	75.807	71.51604	66.489	72.27065	75.68935	6.585698	128.233	126.9634	134.195	132.8663	130.388	131.7051	130.5116	3.127225
8	77.083	75.57157	77.954	73.54151	52.625	57.20109	68.77139	10.07145	119.526	118.3426	115.28	114.1386	119.488	120.6949	117.7254	3.321458
9	71.735	70.32843	57.617	54.35566	43.749	47.55326	57.41245	11.69124	92.626	91.70891	90.659	89.76139	84.739	85.59495	89.02175	3.123368
10	74.076	72.62353	79.151	74.67075	58.648	63.74783	70.34737	5.806313	120.382	119.1901	108.319	107.2465	112.053	113.1848	113.2072	5.971813
11	73.95	72.5	53.731	50.68962	48.069	52.24891	58.47951	12.1671	83.946	83.11485	81.704	80.89505	78.781	79.57677	81.19556	1.788082
12	80.355	78.77941	67.405	63.58962	52.202	56.7413	66.37011	11.27909	102.16	101.1485	103.63	102.604	104.16	105.2121	102.9882	2.058872

Cell #	Sneu28							
	S1S1	Adjusted	S1S2	Adjusted	S1S3	Adjusted	Average	SD
1	39.225	40.85938	40.345	38.79327			39.82632	1.460957
2	42.112	43.86667	51.951	49.95288			46.90978	4.303606
3	39.627	41.27813	37.934	36.475			38.87656	3.396322
4	45.158	47.03958	41.941	40.32788			43.68373	4.745888
5	70.657	73.60104	57.745	55.52404			64.56254	12.78237
6	52.995	55.20313	61.124	58.77308			56.9881	2.524337
7	50.732	52.84583	45.321	43.57788			48.21186	6.553429
8	59.915	62.41146	59.768	57.46923			59.94034	3.494683
9	60.127	62.63229	50.776	48.82308			55.72768	9.764589
10	92.769	96.63438	92.173	88.62788			92.63113	5.661444
11	71.67	74.65625	55.723	53.57981			64.11803	14.9033
12	54.742	57.02292	45.05	43.31731			50.17011	9.691329

Type II (ox)

Cell #	Sneu26								Sneu27							
	S1S1	Adjusted	S1S2	Adjusted	S1S3	Adjusted	Average	SD	S1S1	Adjusted	S1S2	Adjusted	S1S3	Adjusted	Average	SD
1	211.177	207.0363	204.314	192.7491	187.135	203.4076	201.0643	7.426266	194.959	193.0287	196.341	194.397	197.649	199.6455	195.6904	3.492838
2	201.238	197.2922	206.377	194.6953	184.737	200.8011	197.5962	3.064234	206.984	204.9347	202.427	200.4228	199.743	201.7606	202.3727	2.317378
3	229.225	224.7304	220.541	208.0575	188.471	204.8598	212.5492	10.66967	208.228	206.1663	206.673	204.6267	194.234	196.196	202.3297	5.367444
4	209.231	205.1284	201.345	189.9481	172.158	187.1283	194.0683	9.681596	213.925	211.8069	219.147	216.9772	209.653	211.7707	213.5183	2.995584
5	201.571	197.6186	210.652	198.7283	200.843	218.3076	204.8848	11.63769	210.413	208.3297	207.788	205.7307	202.499	204.5444	206.2016	1.936071
6	211.357	207.2127	203.967	192.4217	180.764	196.4826	198.7057	7.64201	183.644	181.8257	198.878	196.9089	196.604	198.5899	192.4415	9.231871
7	213.143	208.9637	205.509	193.8764	175.075	190.2989	197.713	9.906233	209.382	207.3089	205.471	203.4366	198.278	200.2808	203.6755	3.520132
8	204.474	200.4647	209.022	197.1906	183.57	199.5326	199.0626	1.686908	213.868	211.7505	218.583	216.4188	202.603	204.6495	210.9396	5.926413
9	191.823	188.0618	191.063	180.2481	181.651	197.4467	188.5855	8.611268	211.527	209.4327	198.722	196.7545	196.591	198.5768	201.588	6.854545
10	193.327	189.5363	197.095	185.9387	174.491	189.6641	188.3797	2.114948	209.747	207.6703	210.702	208.6158	199.318	201.3313	205.8725	3.961084
11	189.886	186.1627	210.506	198.5906	187.183	203.4598	196.071	8.919525	215.187	213.0564	221.341	219.1495	215.408	217.5838	216.5966	3.164232
12	210.003	205.8853	211.923	199.9274	183.299	199.238	201.6836	3.65509	199.767	197.7891	194.304	192.3802	183.377	185.2293	191.7995	6.30001

Cell #	Sneu28							
	S1S1	Adjusted	S1S2	Adjusted	S1S3	Adjusted	Average	SD
1	165.295	172.1823	162.139	155.9029			164.0426	11.51128
2	207.654	216.3063	148.622	142.9058			179.606	51.90198
3	161.895	168.6406	140.826	135.4096			152.0251	23.49787
4	176.261	183.6052	x				183.6052	
5	154.647	161.0906	x				161.0906	
6	158.695	165.3073	x				165.3073	
7	185.162	192.8771	170.456	163.9			178.3885	20.48989
8	207.133	215.7635	220.198	211.7288			213.7462	2.852961
9	216.207	225.2156	192.244	184.85			205.0328	28.54281
10	219.288	228.425	215.99	207.6827			218.0538	14.66703
11	203.721	212.2094	x				212.2094	
12	180.763	188.2948	203.5	195.6731			191.9839	5.217236

mtDNA mutator (#21-4410): SDH and NBTx

Adjustment Factor:

	Azide	AF	NBTx	AF
S1	111	0.979412	18.79	1.06
S2	116	1.023529	15.95	0.90
S3	113	0.997059	18.69	1.05
	113.3333		17.81	

GA

Type II (ox)

Cell #	Azide-S1	Adjusted	Azide-S2	Adjusted	Azide-S3	Adjusted	Average Azide		NBTx-S1	Adjusted	NBTx-S2	Adjusted	NBTx-S3	Adjusted	Average NBTx	
1	206.6	210.8163	183.39	179.7941176	184.67	185.2257	191.9454	16.56684	70.147	66.17642	91.009	101.1211	136.636	130.1295	99.14235	32.02244
2	210.214	214.5041	187.579	183.9009804	180.863	181.4072	193.2708	18.43082	53.303	50.28585	60.554	67.28222	86.515	82.39524	66.65444	16.0639
3	203.725	207.8827	168.944	165.6313725	163.821	164.3139	179.276	24.78285	48.364	45.62642	84.757	94.17444	81.843	77.94571	72.58219	24.71444
4	208.63	212.8878	196.113	192.2676471	195.783	196.3721	200.5092	10.91484	58.64	55.32075	53.062	58.95778	86.581	82.4581	65.57888	14.73051
5	197.831	201.8684	194.105	190.2990196	187.759	188.324	193.4971	7.316662	31.84	30.03774	42.32	47.02222	53.927	51.35905	42.80634	11.26853
6	184.789	188.5602	189.012	185.3058824	195.551	196.1394	190.0018	5.558786	25.919	24.45189	37.627	41.80778	42.293	40.27905	35.5129	9.60957
7	182.005	185.7194	153.28	150.2745098			167.9969	25.06331	35.004	33.02264	41.362	45.95778	60.507	57.62571	45.53538	12.30697
8	210.527	214.8235	225.636	221.2117647	217.5	218.1545	218.0632	3.195125	57.94	54.66038	48.367	53.74111	93.029	88.59905	65.66685	19.86519
9	183.354	187.0959	194.101	190.295098	194.889	195.4754	190.9555	4.228607	33.045	31.17453	15.595	17.32778	43.715	41.63333	30.04521	12.19207
10	209.826	214.1082			170.835	171.349	192.7286	30.23526	98.988	93.38491	143.984	159.9822	166.912	158.9638	137.4436	38.15939
11	212.327	216.6602	211.295	207.1519608	220.897	221.5617	215.1246	7.326565	54.535	51.44811	55.22	61.35556	66.56	63.39048	58.73138	6.389032
12	209.333	213.6051	198.589	194.695098	207.519	208.1434	205.4812	9.732041	65.46	61.75472	74.948	83.27556	67.539	64.32286	69.78438	11.75405
13	207.846	212.0878	204.843	200.8264706	208.271	208.8977	207.2706	5.804275	60.327	56.91226	49.623	55.13667	52.657	50.14952	54.06615	3.506161
14	213.362	217.7163	208.506	204.4176471	224.911	225.5878	215.9072	10.70038	64.686	61.02453	47.962	53.29111	50.681	48.26762	54.19442	6.426248
15	212.217	216.548	216.872	212.6196078	227.774	228.4594	219.209	8.248355	104.659	98.73491	68.026	75.58444	55.797	53.14	75.81978	22.79836

Type II (gly)

Cell #	Azide-S1	Adjusted	Azide-S2	Adjusted	Azide-S3	Adjusted	Average Azide		NBTx-S1	Adjusted	NBTx-S2	Adjusted	NBTx-S3	Adjusted	Average NBTx	
1	25.876	26.40408	29.549	28.96960784	30.555	30.86364	28.74578	2.238187	1.144	1.079245	2.524	2.804444	1.427	1.359048	1.747579	0.925903
2	65.945	67.29082	75.36	73.88235294	71.152	71.87071	71.01463	3.378128	3.206	3.024528	2.859	3.176667	7.268	6.921905	4.374367	2.207544
3	28.126	28.7	22.465	22.0245098	18.637	18.82525	23.18325	5.038321	0.866	0.816981	1.597	1.774444	2.616	2.491429	1.694285	0.840097
4	23.724	24.20816	24.204	23.72941176	22.875	23.10606	23.68121	0.55263	0.398	0.375472	0.987	1.096667	2.974	2.832381	1.43484	1.262882
5	63.689	64.98878	61.104	59.90588235	69.643	70.34646	65.08037	5.220894	2.248	2.120755	2.271	2.523333	4.834	4.60381	3.082633	1.332667
6	33.762	34.45102	28.23	27.67647059	26.028	26.29091	29.4728	4.366572	1.024	0.966038	0.599	0.665556	2.082	1.982857	1.204817	0.69035
7	30.248	30.86531	27.407	26.86960784	22.135	22.35859	26.69783	4.255961	1.21	1.141509	0.395	0.438889	1.923	1.831429	1.137276	0.696279
8	47.083	48.04388			54.354	54.90303	51.47345	4.850153	2.141	2.019811	6.698	7.442222	4.142	3.944762	4.468932	2.748945
9	50.291	51.31735	71.492	70.09019608	75.215	75.97475	65.7941	12.87786	3.023	2.851887	5.327	5.918889	6.924	6.594286	5.121687	1.994501
10	40.096	40.91429	42.882	42.04117647	61.176	61.79394	48.2498	11.74309	1.432	1.350943	1.299	1.443333	2.946	2.805714	1.866664	0.814553
11	80.989	82.64184	74.711	73.24607843	89.788	90.69495	82.19429	8.733041	4.625	4.363208	6.129	6.81	9.333	8.888571	6.68726	2.265177
12	75.26	76.79592	83.328	81.69411765	96.225	97.19697	85.229	10.64999	4.984	4.701887	5.63	6.255556	6.703	6.38381	5.780417	0.936234
13	81.728	83.39592	79.606	78.04509804	98.186	99.17778	86.87293	10.98703	6.921	6.529245	6.231	6.923333	4.271	4.067619	5.840066	1.547579
14	32.875	33.54592	36.008	35.30196078	41.651	42.07172	36.9732	4.501898	1.83	1.726415	1.673	1.858889	2.584	2.460952	2.015419	0.391488
15	72.144	73.61633	74.209	72.75392157	86.204	87.07475	77.815	8.030763	6.296	5.939623	2.509	2.787778	4.627	4.406667	4.378022	1.576118

Soleus

Type I

Cell #	Azide-S1	Adjusted	Azide-S2	Adjusted	Azide-S3	Adjusted	Average Azide		NBTx-S1	Adjusted	NBTx-S2	Adjusted	NBTx-S3	Adjusted	Average NBTx	
1	161.713	165.0133	140.504	137.7490196	159.297	159.7763	154.1795	14.46817	38.717	36.52547	18.477	20.53	16.683	15.88857	24.31468	10.82651
2	182.815	186.5459	174.455	171.0343137	191.725	192.3019	183.294	11.00039	78.523	74.0783	48.818	54.24222	35.78	34.07619	54.13224	20.00128
3	161.568	164.8653	143.237	140.4284314	167.465	167.9689	157.7542	15.0846	45.545	42.96698	31.681	35.20111	24.137	22.98762	33.71857	10.07185
4	178.195	181.8316	166.262	163.0019608	195.63	196.2187	180.3507	16.65779	50.06	47.22642	33.417	37.13	22.996	21.90095	35.41912	12.74912
5	157.889	161.1112	153.834	150.8176471	177.422	177.9559	163.2949	13.70026	35.849	33.81981	16.698	18.55333	14.69	13.99048	22.12121	10.38499
6	148.749	151.7847	143.238	140.4294118	170.584	171.0973	154.4371	15.50504	44.376	41.86415	20.275	22.52778	17.087	16.27333	26.88842	13.34106
7	165.728	169.1102	161.629	158.4598039	179.025	179.5637	169.0446	10.5521	39.42	37.18868	19.895	22.10556	13.956	13.29143	24.19522	12.08489
8	149.296	152.3429	143.552	140.7372549	167.514	168.0181	153.6994	13.6909	39.45	37.21698	28.718	31.90889	16.931	16.12476	28.41688	10.97115
9	168.962	172.4102	170.4	167.0588235	205.75	206.3691	181.946	21.31956	32.227	30.40283	8.962	9.957778	9.816	9.348571	16.56973	11.98369
10	141.136	144.0163	156.078	153.0176471	164.398	164.8927	153.9756	10.47109	28.911	27.27453	21.969	24.41	12.263	11.67905	21.12119	8.301623
11	169.493	172.952	155.235	152.1911765	179.369	179.9087	168.3506	14.42031	44.624	42.09811	25.912	28.79111	29.273	27.87905	32.92276	7.959167
12	190.08	193.9592	184.995	181.3676471	221.855	222.5226	199.2831	21.08768	45.586	43.00566	63.41	70.45556	77.758	74.05524	62.50548	16.98299
13	137.545	140.352	132.032	129.4431373	168.089	168.5948	146.13	20.20523	41.545	39.1934	27.543	30.60333	24.807	23.62571	31.14081	7.797746
14	171.138	174.6306	164.671	161.4421569	193.126	193.7071	176.5933	16.22178	48.545	45.79717	25.537	28.37444	28.66	27.29524	33.82228	10.38458
15	172.655	176.1786	171.995	168.622549	190.221	190.7934	178.5315	11.27114	56.085	52.91038	36.397	40.44111	37.313	35.53619	42.96256	8.957336

mtDNA mutator (#21-4335): SDH and NBTx

Adjustment Factor

	Azide	AF	NBTx	AF
S1	88.052	0.97059796	11.491	0.72
S2	105.841	1.16668626	26.409	1.663733725
S3	78.265	0.86271578	9.72	0.612347753
	90.71933		15.8733333	

GA

Type II (ox)

Cell #	Azide-S1	Adjusted	Azide-S2	Adjusted	Azide-S3	Adjusted	Average Azide		NBTx-S1	Adjusted	NBTx-S2	Adjusted	NBTx-S3	Adjusted	Average NBTx	
1	206.61	213	241.896	206.7487179	198.583	230.1877825	216.6455	12.13733	30.038	41.719444	76.769	46.246386	28.799	47.211475	45.059102	2.932206
2	205.04	211.381443	229.078	195.7931624	201.304	233.3418338	213.5055	18.86423	54.221	75.306944	94.563	56.965663	48.691	79.821311	70.697973	12.10484
3	217.932	224.672165	240.086	205.2017094	205.448	238.1453576	222.6731	16.56256	32.871	45.654167	71.677	43.178916	32.561	53.378689	47.403924	5.320252
4	219.318	226.101031	245.778	210.0666667	203.625	236.0322244	224.0666	13.10178	58.091	80.681944	103.221	62.181325	52.2	85.57377	76.14568	12.33835
5	205.724	212.086598	223.593	191.1051282	195.861	227.0325722	210.0748	18.04802	52.623	73.0875	95.99	57.825301	39.998	65.570492	65.494431	7.631384
6	199.285	205.448454	217.911	186.2487179	200.844	232.8086241	208.1686	23.39884	37.102	51.530556	83.025	50.01506	28.43	46.606557	49.384058	2.521916
7	207.871	214.3	230.215	196.7649573	202.458	234.6794946	215.2482	18.97504	53.61	74.458333	99.34	59.843373	42.344	69.416393	67.906033	7.423621
8	208.938	215.4	227.154	194.1487179	210.905	244.4708473	218.0065	25.26212	36.762	51.058333	87.472	52.693976	32.254	52.87541	52.20924	1.000834
9	202.891	209.165979	224.111	191.5478632	203.188	235.5256752	212.0798	22.13323	12.357	17.1625	45.551	27.440361	15.059	24.686885	23.096582	5.320283
10	213.087	219.67732	233.671	199.7188034	195.284	226.3637417	215.2533	13.86244	23.176	32.188889	67.496	40.660241	22.32	36.590164	36.479765	4.236755
11	204.941	211.279381	218.793	187.0025641	188.933	219.0019706	205.7613	16.69812	12.954	17.991667	55.45	33.403614	7.058	11.570492	20.988591	11.22085
12	195.567	201.615464	223.214	190.7811966	177.582	205.8444419	199.4137	7.769245	20.34	28.25	57.151	34.428313	17.762	29.118033	30.598782	3.34475
13	208.281	214.72268	222.497	190.1683761	186.098	215.7157761	206.8689	14.47164	40.545	56.3125	84.527	50.91988	32.794	53.760656	53.664345	2.6976
14	206.52	212.907216	236.447	202.091453	205.731	238.4733975	217.824	18.68268	39.062	54.252778	87.379	52.637952	32.658	53.537705	53.476145	0.809171
15	218.042	224.785567	233.706	199.7487179	207.498	240.5216182	221.6853	20.56249	45.522	63.225	91.41	55.066265	37.641	61.706557	59.999274	4.33905

Type II (gly)

Cell #	Azide-S1	Adjusted	Azide-S2	Adjusted	Azide-S3	Adjusted	Average Azide		NBTx-S1	Adjusted	NBTx-S2	Adjusted	NBTx-S3	Adjusted	Average NBTx	
1	48.921	50.4340206	51.699	44.18717949	34.992	40.68837209	45.10319	4.936975	6.248	8.6777778	8.304	5.0024096	2.225	3.647541	5.7759095	2.602796
2	51.725	53.3247423	57.217	48.9034188	33.379	38.8127907	47.01365	7.438253	5.623	7.8097222	7.746	4.6662651	3.109	5.0967213	5.8575695	1.704259
3	46.956	48.4082474	51.24	43.79487179	32.435	37.71511628	43.30608	5.363297	3.834	5.325	7.424	4.4722892	2.252	3.6918033	4.4963641	0.816864
4	25.339	26.1226804	28.511	24.36837607	18.408	21.40465116	23.96524	2.38471	2.016	2.8	2.976	1.7927711	2.307	3.7819672	2.7915794	0.994625
5	37.709	38.8752577	56.853	48.59230769	28.018	32.57906977	40.01555	8.067288	2.608	3.6222222	6.632	3.9951807	1.407	2.3065574	3.3079868	0.887085
6	34.71	35.7835052	47.029	40.1957265	35.397	41.15930233	39.04618	2.866338	3.282	4.5583333	8.777	5.2873494	2.493	4.0868852	4.6441893	0.60482
7	34.684	35.756701	46.015	39.32905983	27.908	32.45116279	35.84564	3.439811	6.163	8.5597222	7.912	4.7662651	1.51	2.4754098	5.2671324	3.072925
8	36.734	37.8701031	69.512	59.41196581	34.056	39.6	45.62736	11.96912	1.749	2.4291667	2.308	1.3903614	3.435	5.6311475	3.1502252	2.210432
9	48.097	49.5845361	60.545	51.74786325	33.788	39.28837209	46.87359	6.657451	2.238	3.1083333	7.19	4.3313253	0.815	1.3360656	2.9252414	1.506
10	60.753	62.6319588	57.625	49.25213675	62.013	72.10813953	61.33075	11.48343	2.01	2.7916667	6.619	3.9873494	2.694	4.4163934	3.7318032	0.841969
11	65.071	67.0835052	63.448	54.22905983	65.942	76.67674419	65.99644	11.26326	4.439	6.1652778	6.878	4.1433735	2.162	3.5442623	4.6176379	1.373363
12	35.448	36.5443299	28.247	24.14273504	19.939	23.18488372	27.95732	7.451978	2.831	3.9319444	2.557	1.5403614	1.945	3.1885246	2.8869435	1.223981
13	36.516	37.6453608	34.302	29.31794872	27.243	31.67790698	32.88041	4.291963	2.367	3.2875	4.367	2.6307229	3.048	4.9967213	3.6383147	1.221389
14	31.249	32.2154639	35.053	29.95982906	26.77	31.12790698	31.10107	1.128057	2.242	3.1138889	4.757	2.8656627	1.536	2.5180328	2.8325281	0.299307
15	37.829	38.9989691	51.281	43.82991453	40.527	47.1244186	43.31777	4.086864	1.342	1.8638889	6.474	3.9	1.191	1.952459	2.572116	1.150834

Soleus

Type I

Cell #	Azide-S1	Adjusted	Azide-S2	Adjusted	Azide-S3	Adjusted	Average Azide		NBTx-S1	Adjusted	NBTx-S2	Adjusted	NBTx-S3	Adjusted	Average NBTx	
1	137.759	142.019588	163.596	139.825641	112.439	130.7430233	137.5294	5.978688	15.19	21.097222	33.259	20.035542	10.034	16.44918	19.193982	2.435619
2	147.419	151.978351	165.757	141.6726496	135.589	157.6616279	150.4375	8.105086	6.736	9.3555556	33.378	20.107229	7.88	12.918033	14.126939	5.476834
3	142.525	146.93299	161.936	138.4068376	131.884	153.3534884	146.2311	7.498005	11.471	15.931944	34.804	20.966265	7.304	11.97377	16.29066	4.506967
4	142.303	146.704124	155.145	132.6025641	121.11	140.8255814	140.0441	7.083187	12.726	17.675	33.173	19.983735	6.533	10.709836	16.122857	4.827853
5	154.272	159.043299	167.89	143.4957265	131.077	152.4151163	151.6514	7.801873	20.549	28.540278	43.792	26.380723	17.55	28.770492	27.897164	1.318311
6	161.588	166.585567	162.237	138.6641026	126.617	147.2290698	150.8262	14.30408	14.483	20.115278	36.282	21.856627	11.165	18.303279	20.091728	1.776791
7	140.427	144.770103	181.096	154.782906	140.021	162.8151163	154.1227	9.040604	27.984	38.866667	53.934	32.490361	20.561	33.706557	35.021195	3.385339
8	152.47	157.185567	169.819	145.1444444	144.516	168.0418605	156.7906	11.45382	17.228	23.927778	40.168	24.19759	11.709	19.195082	22.44015	2.813548
9	172.256	177.583505	187.088	159.9042735	145.088	168.7069767	168.7316	8.839642	8.438	11.719444	43.806	26.389157	8.464	13.87541	17.328004	7.920885
10	151.181	155.856701	175.577	150.065812	118.453	137.7360465	147.8862	9.254869	11.057	15.356944	35.457	21.359639	11.972	19.62623	18.780938	3.089332
11	132.974	137.086598	169.078	144.5111111	112.886	131.2627907	137.6202	6.640258	17.576	24.411111	42.419	25.553614	18.895	30.97541	26.980045	3.506926
12	139.923	144.250515	171.741	146.7871795	98.062	114.0255814	135.0211	18.22683	20.596	28.605556	53.106	31.991566	13.573	22.25082	27.61598	4.945198
13	100.722	103.837113	135.659	115.9478632	94.102	109.4209302	109.7353	6.061492	11.387	15.815278	36.161	21.783735	6.781	11.116393	16.238469	5.346247
14	129.765	133.778351	154.241	131.8299145	136.75	159.0116279	141.54	15.16224	12.299	17.081944	33.034	19.9	10.048	16.472131	17.818025	1.828642
15	139.85	144.175258	161.918	138.391453	141.828	164.9162791	149.161	13.94757	11.838	16.441667	30.99	18.668675	4.388	7.1934426	14.101261	6.085093

mtDNA mutator (#21-4336): SDH and NBTx

Adjustment Factor

	Azide	AF	NBTx	AF
S1	86.368	0.864706	29.114	1.06
S2	109.16	1.092897	25.683	0.937387083
S3	104.116	1.042397		
	99.88133		27.3985	

GA

Type II (ox)

Cell #	Azide-S1	Adjusted	Azide-S2	Adjusted	Azide-S3	Adjusted	Average Azide		NBTx-S1	Adjusted	NBTx-S2	Adjusted	NBTx-S3	Adjusted	Average NBTx	
1	218.49	254.0581	236.834	217.2788991	222.554	213.9942	228.4438	22.24342	135.489	127.8198	114.139	121.4245			124.6221	4.522191
2	200.719	233.3942	233.975	214.6559633	214.499	206.249	218.0997	13.89638	61.725	58.23113	73.068	77.73191			67.98152	13.78914
3	162.974	189.5047	181.477	166.4926606	165.934	159.5519	171.8497	15.6785	41.503	39.15377	26.799	28.50957			33.83167	7.526585
4	196.74	228.7674	233.625	214.3348624	215.721	207.424	216.8421	10.89036	87.267	82.32736	44.748	47.60426			64.96581	24.55294
5	208.234	242.1326	240.982	221.0844037	185.788	178.6423	213.9531	32.3403	84.56	79.77358	40.682	43.27872			61.52615	25.80576
6	196.999	229.0686	230.2	211.1926606	215.616	207.3231	215.8614	11.60022	51.658	48.73396	38.323	40.76915			44.75156	5.631974
7	229.211	266.5244	233.997	214.6761468	235.089	226.0471	235.7492	27.25177	84.231	79.46321	79.091	84.13936			81.80128	3.30654
8	226.325	263.1686	231.809	212.6688073	242.432	233.1077	236.315	25.40222	72.849	68.72547	67.83	72.15957			70.44252	2.428277
9	215.454	250.5279	236.716	217.1706422	226.938	218.2096	228.6361	18.96602	97.542	92.02075	56.938	60.57234			76.29655	22.23739
10	201.365	234.1453	230.646	211.6018349	221.388	212.8731	219.5401	12.66449	61.471	57.99151	52.672	56.03404			57.01278	1.384138
11	191.662	222.8628	225.323	206.7183486	199.684	192.0038	207.195	15.43499	74.788	70.55472	50.816	54.05957			62.30715	11.66383
12	205.687	239.1709	231.49	212.3761468	234.754	225.725	225.7574	13.39742	64.176	60.5434	66.029	70.24362			65.39351	6.859092
13	189.91	220.8256	226.364	207.6733945	224.576	215.9385	214.8125	6.647998	68.443	64.56887	59.977	63.80532			64.18709	0.539911
14	193.389	224.8709	226.13	207.4587156	224.695	216.0529	216.1275	8.706347	55.415	52.2783	50.153	53.35426			52.81628	0.760814
15	193.913	225.4802	207.826	190.666055			208.0731	24.61734	27.412	25.86038	23.316	24.80426			25.33232	0.746791

Type II (gly)

Cell #	Azide-S1	Adjusted	Azide-S2	Adjusted	Azide-S3	Adjusted	Average Azide		NBTx-S1	Adjusted	NBTx-S2	Adjusted	NBTx-S3	Adjusted	Average NBTx	
1	67.043	77.95698	94.159	86.38440367	81.184	78.06154	80.80097	4.835675	18.468	17.42264	7.068	7.519149			12.4709	7.002827
2	68.638	79.81163	96.267	88.31834862	68.411	65.77981	77.96993	11.38158	14.914	14.06981	11.406	12.13404			13.10193	1.368795
3	31.259	36.34767	44.087	40.44678899	39.674	38.14808	38.31418	2.054599	3.275	3.089623	1.816	1.931915			2.510769	0.818623
4	45.61	53.03488	62.815	57.62844037	49.046	47.15962	52.60765	5.247473	4.125	3.891509	4.364	4.642553			4.267031	0.531068
5	33.314	38.73721	42.038	38.56697248	39.84	38.30769	38.53729	0.216291	5.129	4.838679	3.35	3.56383			4.201255	0.901455
6	50.333	58.52674	82.907	76.06146789	77.814	74.82115	69.80312	9.785301	5.917	5.582075	6.198	6.593617			6.087846	0.715268
7	35.09	40.80233	53.991	49.53302752	44.342	42.63654	44.32396	4.60346	6.038	5.696226	4.777	5.081915			5.389071	0.434384
8	50.967	59.26395	81.692	74.94678899	69.743	67.06058	67.09044	7.84146	7.141	6.736792	5.361	5.703191			6.219992	0.730866
9	46.284	53.8186	58.951	54.08348624	61.071	58.72212	55.5414	2.757761	5.459	5.15	8.532	9.076596			7.113298	2.776522
10	24.102	28.02558	31.951	29.31284404	38.127	36.66058	31.333	4.658494	1.426	1.345283	1.81	1.925532			1.635407	0.410298
11	78.011	90.71047	111.733	102.5073394	98.64	94.84615	96.02132	5.985593	14.434	13.61698	15.262	16.23617			14.92658	1.852046
12	42.342	49.23488	64.302	58.99266055	68.616	65.97692	58.06816	8.409221	5.988	5.649057	7.723	8.215957			6.932507	1.815073
13	38.457	44.71744	30.069	27.58623853	45.858	44.09423	38.7993	9.715798	3.82	3.603774	5.251	5.58617			4.594972	1.401766
14	47.514	55.24884	61.513	56.43394495	76.708	73.75769	61.81349	10.36094	4.237	3.99717	4.107	4.369149			4.183159	0.263029
15	38.584	44.86512	49.68	45.57798165	63.753	61.30096	50.58135	9.290293	5.721	5.39717	6.771	7.203191			6.300181	1.27705

Soleus

Type I

Cell #	Azide-S1	Adjusted	Azide-S2	Adjusted	Azide-S3	Adjusted	Average Azide		NBTx-S1	Adjusted	NBTx-S2	Adjusted	NBTx-S3	Adjusted	Average NBTx	
1	134.321	156.1872			150.283	144.5029	150.345	8.262065	42.018	39.63962	59.39	63.18085			51.41024	16.64616
2	139.955	162.7384			143.306	137.7942	150.2663	17.63817	59.084	55.73962	67.47	71.7766			63.75811	11.33985
3	165.782	192.7698			196.317	188.7663	190.7681	2.830846	47.211	44.53868	68.945	73.34574			58.94221	20.36967
4	158.734	184.5744			178.716	171.8423	178.2084	9.002962			59.012	62.77872			62.77872	#DIV/0!
5	157.841	183.536			156.128	150.1231	166.8296	23.62654			30.614	32.56809			32.56809	#DIV/0!
6	159.239	185.1616			166.78	160.3654	172.7635	17.53359	40.526	38.23208	45.768	48.68936			43.46072	7.394418
7	154.693	179.8756			148.912	143.1846	161.5301	25.94443	47.721	45.01981	66.212	70.4383			57.72905	17.97358
8	146.149	169.9407			167.508	161.0654	165.503	6.275794	61.27	57.80189	86.936	92.48511			75.1435	24.52474
9	148.502	172.6767			146.204	140.5808	156.6288	22.69528	59.69	56.31132	77.728	82.68936			69.50034	18.65209
10	168.813	196.2942			157.035	150.9952	173.6447	32.03123	42.347	39.95	58.039	61.74362			50.84681	15.41041
11	141.461	164.4895			167.49	161.0481	162.7688	2.433478	38.564	36.38113	51.917	55.23085			45.80599	13.32876
12	142.251	165.4081			173.682	167.0019	166.205	1.126975	36.677	34.60094	43.111	45.86277			40.23185	7.963311
13	154.946	180.1698			177.432	170.6077	175.3887	6.761408	26.241	24.75566	44.156	46.97447			35.86506	15.71107
14	143.906	167.3326			163.793	157.4933	162.4129	6.957428	19.753	18.63491	32.788	34.88085			26.75788	11.48762
15	141.075	164.0407			159.801	153.6548	158.8478	7.343933	26.751	25.23679	21.328	22.68936			23.96308	1.801306

SDH activity (1 mM azide) as ROD value: Comparing Wild-Type versus Mutator

	Type II (ox)			Type I			Type II (gly)			Type II (ox)			Type I			Type II (gly)		
	Sneu26	Sneu27	Sneu28	Sneu26	Sneu27	Sneu28	Sneu26	Sneu27	Sneu28	4335	4410	4336	4335	4410	4336	4335	4410	4336
	201.0643	195.6904	164.0426	149.6591	176.0523	173.4913	49.63751	116.1396	39.82632	216.6455	191.9454	228.4438	137.5294	154.1795	150.345	43.77345	28.74578	80.80097
	197.5962	202.3727	179.606	133.5357	147.4741	136.787	63.86643	91.89864	46.90978	213.5055	193.2708	218.0997	150.4375	183.294	150.2663	45.63678	71.01463	77.96993
	212.5492	202.3297	152.0251	149.625	171.9494	163.1678	55.32087	83.20962	38.87656	222.6731	179.276	171.8497	146.2311	157.7542	190.7681	42.03327	23.18325	38.31418
	194.0683	213.5183	183.6052	165.2821	169.336	150.2164	74.9604	80.52047	43.68373	224.0666	200.5092	216.8421	140.0441	180.3507	178.2084	23.26044	23.68121	52.60765
	204.8848	206.2016	161.0906	141.6452	150.429	111.9664	53.92229	86.34077	64.56254	210.0748	193.4971	213.9531	151.6514	163.2949	166.8296	38.8558	65.08037	38.53729
	198.7057	192.4415	165.3073	135.7402	191.6556	112.8302	70.91096	117.7143	56.9881	208.1686	190.0018	215.8614	150.8262	154.4371	172.7635	37.89091	29.4728	69.80312
	197.713	203.6755	178.3885	131.5845	169.8504	126.7708	75.68935	130.5116	48.21186	215.2482	167.9969	235.7492	154.1227	169.0446	161.5301	34.79571	26.69783	44.32396
	199.0626	210.9396	213.7462	141.6704	187.7348	131.2647	68.77139	117.7254	59.94034	218.0065	218.0632	236.315	156.7906	153.6994	165.503	44.30861	51.47345	67.09044
	188.5855	201.588	205.0328	143.7235	172.216	143.738	57.41245	89.02175	55.72768	212.0798	190.9555	228.6361	168.7316	181.946	156.6288	45.50507	65.7941	55.5414
	188.3797	205.8725	218.0538	154.3529	173.6409	124.6648	70.34737	113.2072	92.63113	215.2533	192.7286	219.5401	147.8862	153.9756	173.6447	59.48379	48.2498	31.333
	196.071	216.5966	212.2094	150.4687	158.6732	150.4474	58.47951	81.19556	64.11803	205.7613	215.1246	207.195	137.6202	168.3506	162.7688	64.01216	82.19429	96.02132
	201.6836	191.7995	191.9839	134.4399	168.3512	136.5095	66.37011	102.9882	50.17011	199.4137	205.4812	225.7574	135.0211	199.2831	166.205	27.13018	85.229	58.06816
**Average**	**198.3637**	**203.5855**	**185.4243**	**144.3106**	**169.7802**	**138.4879**	**63.80739**	**100.8728**	**55.13718**	**213.4081**	**194.9042**	**218.1869**	**148.0743**	**168.3008**	**166.2884**	**42.22385**	**50.06804**	**59.20095**

Average of 3 replicates (S1 to S3), adjusted.

GA

		21-4335			21-4410			21-4336		
	Cell #	SDH	NBTx	Deficiency	SDH	NBTx	Deficiency	SDH	NBTx	Deficiency
Type II (ox)	1	216.6455	45.059102	20.80%	191.9454	99.14235	51.65%	228.4438	124.6221	54.55%
	2	213.5055	70.697973	33.11%	193.2708	66.65444	34.49%	218.0997	67.98152	31.17%
	3	222.6731	47.403924	21.29%	179.276	72.58219	40.49%	171.8497	33.83167	19.69%
	4	224.0666	76.14568	33.98%	200.5092	65.57888	32.71%	216.8421	64.96581	29.96%
	5	210.0748	65.494431	31.18%	193.4971	42.80634	22.12%	213.9531	61.52615	28.76%
	6	208.1686	49.384058	23.72%	190.0018	35.5129	18.69%	215.8614	44.75156	20.73%
	7	215.2482	67.906033	31.55%	167.9969	45.53538	27.10%	235.7492	81.80128	34.70%
	8	218.0065	52.20924	23.95%	218.0632	65.66685	30.11%	236.315	70.44252	29.81%
	9	212.0798	23.096582	10.89%	190.9555	30.04521	15.73%	228.6361	76.29655	33.37%
	10	215.2533	36.479765	16.95%	192.7286	137.4436	71.31%	219.5401	57.01278	25.97%
	11	205.7613	20.988591	10.20%	215.1246	58.73138	27.30%	207.195	62.30715	30.07%
	12	199.4137	30.598782	15.34%	205.4812	69.78438	33.96%	225.7574	65.39351	28.97%
	13	206.8689	53.664345	25.94%	207.2706	54.06615	26.08%	214.8125	64.18709	29.88%
	14	217.824	53.476145	24.55%	215.9072	54.19442	25.10%	216.1275	52.81628	24.44%
	15	221.6853	59.999274	27.07%	219.209	75.81978	34.59%	208.0731	25.33232	12.17%
	STDV	6.870036	16.815857	7.57%	14.82643	26.52892	13.85%	15.36578	22.61334	9.18%

		21-4335			21-4410			21-4336		
	Cell #	SDH	NBTx	Deficiency	SDH	NBTx	Deficiency	SDH	NBTx	Deficiency
Type II (gly)	1	43.77345	5.7759095	13.20%	28.74578	1.747579	6.08%	80.80097	12.4709	15.43%
	2	45.63678	5.8575695	12.84%	71.01463	4.374367	6.16%	77.96993	13.10193	16.80%
	3	42.03327	4.4963641	10.70%	23.18325	1.694285	7.31%	38.31418	2.510769	6.55%
	4	23.26044	2.7915794	12.00%	23.68121	1.43484	6.06%	52.60765	4.267031	8.11%
	5	38.8558	3.3079868	8.51%	65.08037	3.082633	4.74%	38.53729	4.201255	10.90%
	6	37.89091	4.6441893	12.26%	29.4728	1.204817	4.09%	69.80312	6.087846	8.72%
	7	34.79571	5.2671324	15.14%	26.69783	1.137276	4.26%	44.32396	5.389071	12.16%
	8	44.30861	3.1502252	7.11%	51.47345	4.468932	8.68%	67.09044	6.219992	9.27%
	9	45.50507	2.9252414	6.43%	65.7941	5.121687	7.78%	55.5414	7.113298	12.81%
	10	59.48379	3.7318032	6.27%	48.2498	1.866664	3.87%	31.333	1.635407	5.22%
	11	64.01216	4.6176379	7.21%	82.19429	6.68726	8.14%	96.02132	14.92658	15.55%
	12	27.13018	2.8869435	10.64%	85.229	5.780417	6.78%	58.06816	6.932507	11.94%
	13	31.9037	3.6383147	11.40%	86.87293	5.840066	6.72%	38.7993	4.594972	11.84%
	14	30.1795	2.8325281	9.39%	36.9732	2.015419	5.45%	61.81349	4.183159	6.77%
	15	42.03304	2.572116	6.12%	77.815	4.378022	5.63%	50.58135	6.300181	12.46%
	STDV	10.99432	1.1263675	2.89%	24.12554	1.931816	1.49%	18.36032	3.880169	3.48%

Soleus

		21-4335			21-4410			21-4336		
	Cell #	SDH	NBTx	Deficiency	SDH	NBTx	Deficiency	SDH	NBTx	Deficiency
Type I	1	137.5294	19.193982	13.96%	154.1795	24.31468	15.77%	150.345	51.41024	34.19%
	2	150.4375	14.126939	9.39%	183.294	54.13224	29.53%	150.2663	63.75811	42.43%
	3	146.2311	16.29066	11.14%	157.7542	33.71857	21.37%	190.7681	58.94221	30.90%
	4	140.0441	16.122857	11.51%	180.3507	35.41912	19.64%	178.2084	62.77872	35.23%
	5	151.6514	27.897164	18.40%	163.2949	22.12121	13.55%	166.8296	32.56809	19.52%
	6	150.8262	20.091728	13.32%	154.4371	26.88842	17.41%	172.7635	43.46072	25.16%
	7	154.1227	35.021195	22.72%	169.0446	24.19522	14.31%	161.5301	57.72905	35.74%
	8	156.7906	22.44015	14.31%	153.6994	28.41688	18.49%	165.503	75.1435	45.40%
	9	168.7316	17.328004	10.27%	181.946	16.56973	9.11%	156.6288	69.50034	44.37%
	10	147.8862	18.780938	12.70%	153.9756	21.12119	13.72%	173.6447	50.84681	29.28%
	11	137.6202	26.980045	19.60%	168.3506	32.92276	19.56%	162.7688	45.80599	28.14%
	12	135.0211	27.61598	20.45%	199.2831	62.50548	31.37%	166.205	40.23185	24.21%
	13	109.7353	16.238469	14.80%	146.13	31.14081	21.31%	175.3887	35.86506	20.45%
	14	141.54	17.818025	12.59%	176.5933	33.82228	19.15%	162.4129	26.75788	16.48%
	15	149.161	14.101261	9.45%	178.5315	42.96256	24.06%	158.8478	23.96308	15.09%
	STDV	13.12022	6.0958025	4.16%	14.94528	12.43878	5.92%	10.79155	15.53011	9.80%
